# Use of the visual analogue scale for health state valuation: a scoping review

**DOI:** 10.1007/s11136-023-03411-3

**Published:** 2023-04-07

**Authors:** Mimmi Åström, Zin Min Thet Lwin, Fitsum Sebsibe Teni, Kristina Burström, Jenny Berg

**Affiliations:** 1 Health Outcomes and Economic Evaluation Research Group, Stockholm Centre for Healthcare Ethics, Department of Learning, Informatics, Management and Ethics, Karolinska Institutet, Tomtebodavägen 18 A, 171 77 Stockholm, Sweden; 2 Equity and Health Policy Research Group, Department of Global Public Health, Karolinska Institutet, Stockholm, Sweden; 3 Centre for Health Economics, Informatics and Health Services Research, Stockholm Health Care Services, Region Stockholm, Stockholm, Sweden

**Keywords:** Anchoring, Economic evaluation, Health-related quality of life, Health state valuation, Scoping review, Visual analogue scale

## Abstract

**Objectives:**

The visual analogue scale (VAS) has been used in the context of health and healthcare for various purposes, for example, to measure pain and to provide a single-index measure of health-related quality of life (HRQoL). This scoping review aims to describe how the VAS has been used for health state valuation in the published literature.

**Methods:**

The search was carried out in Medline, Web of Science and PsycInfo. The findings of the included articles were tabulated and presented descriptively using frequencies and proportions.

**Results:**

The database search yielded 4856 unique articles, out of these, 308 were included. In 83% of the articles, the main purpose for using a VAS was to value health states. The two most common perspectives when valuing health states with a VAS were hypothetical (44%) and own health (34%). Some (n = 14) articles used the VAS in the context of economic evaluations, including calculating quality-adjusted life years (QALYs). A large variation in the design of the VAS was found, including the description of the lower and upper anchors. Advantages and disadvantages with using a VAS were mentioned in 14% of the included articles.

**Conclusion:**

The VAS has been a common method for valuing health states, both as a stand-alone method and in combination with other valuation methods. Despite its widespread use, the design of the VAS has been inconsistent which makes comparison of results across studies challenging. Further research on the role of using the VAS in economic evaluations is warranted.

**Supplementary Information:**

The online version contains supplementary material available at 10.1007/s11136-023-03411-3.

## Plain English summary

In the context of healthcare, the visual analogue scale (VAS) is one of the most used valuation methods to provide a single-index measure of health-related quality of life. However, the VAS has been used differently in valuation studies, and the discussion on whether VAS valuations are appropriate for use in cost-utility analyses is ongoing. This study provides a scoping literature review of how the VAS has been used for health state valuation over the last three decades. It shows how the VAS has been used for health state valuation in the published literature and sheds light on some of the advantages and disadvantages of using VAS as a valuation method for informing decisions in healthcare. This provides valuable input to the discussion regarding use of VAS for health state valuation, including use in economic evaluations. In future studies, transparency regarding the design of the VAS including the endpoints is warranted.

## Introduction

Health state values can be elicited through different methods, framed under certainty or uncertainty and based on scaling or choice [1–3]. The most widely used methods are Standard Gamble (SG), Time Trade-Off (TTO), rating scale, including the visual analogue scale (VAS) as one of its variants, and Discrete Choice Experiment (DCE) [1–3]. Different valuation methods have yielded different values for the same health state, and the relationship has been shown to be affected by the severity of the health state [4, 5]. Transformation of VAS valuations to SG and TTO values have also been discussed in different studies, demonstrating the possibility of mapping of VAS scores to SG and TTO scores [5–7].

Generally, choice-based methods, such as SG and TTO, are preferred by many health economists over rating scales, since choosing is considered to be a natural task for humans that is observable and verifiable [1–3]. Rating scales are also claimed to be subject to measurement bias and to not possess interval scale properties [1, 3]. However, other practical aspects weigh in favour of scaling methods, such as reduced amount of time required than for other methods as well as high response and completion rates [1, 3].

The VAS has been used in the context of health and healthcare for various purposes; to measure symptoms (e.g. pain) or different domains of health (e.g. mobility), and to provide a single-index measure of health-related quality of life (HRQoL) [1]. It is also used in economic evaluation as a valuation method, by directly asking individuals about their own health, or as a means of valuing health state classifications, including Quality of Well-being Scale (QWB), Health Utility Index (HUI), 15-D and EQ-5D [1].

There are shortcomings with all valuation methods. While, as an example, challenges with using the SG and TTO for mild health states, temporary health states and for children’s health states have been shown in previous literature [8–10], little is known about related concerns with VAS. There is a lack of a comprehensive overview of how the VAS is used in health state valuation, its purpose, design, benefits and drawbacks. This scoping review aims to describe how the VAS has been used for health state valuation in the published literature. Specifically, we address the following questions:

- What are the overall purposes of using a VAS for health state valuation?
- How have the health states valued using a VAS been defined?
- What designs or forms of the VAS have been used for health state valuation?
- What are the advantages and disadvantages of using a VAS for health state valuation mentioned in the identified articles?

## Methods

Prior to data extraction the protocol was registered at the International prospective register of systematic reviews (PROSPERO) (number CRD42020210041). The review is presented following the Preferred Reporting Items for Systematic Reviews and Meta-Analyses extension for Scoping Reviews (PRISMA-ScR) checklist [11].

### Information sources and search strategy

The search for relevant studies was carried out in Medline (OVID), Web of Science (Clarivate) and PsycInfo (OVID), June 16th, 2020; with an updated search January 27th, 2022, no date limits were applied.

Two blocks of search terms were used: ‘visual analogue scale’ and ‘health state valuation’. A search strategy was developed by the authors and a librarian, were limited to the English language and studies published in peer reviewed journals. The index terms were Medical Subject Headings (MeSH). The full search strategy for each database is presented in Online Resource Table S1.

### Selection criteria and screening process

Inclusion criteria were studies where a VAS was used for health state valuation, and studies where a specific dimension or disease stage were valued using VAS. It was not sufficient for a study to solely report on current health using a VAS. Exclusion criteria were studies where the endpoints for the VAS were specified for a specific condition or disease, if a comparison of only secondary data was performed (e.g. comparisons of value sets), if the publication was a study protocol or a mapping study (e.g. mapping from a condition-specific instrument to a generic instrument).

Titles and abstracts of all identified studies were independently screened for relevance by two reviewers (M.Å. and F.S.T.) using the software Rayyan [12]. In the case of disagreement, the titles and abstracts were read again and discussed among the two reviewers. If not reaching consensus, the studies were included for full-text screening. Authors K.B. and J.B. were engaged in the discussion. All studies meeting the inclusion criteria were used for data extraction, and there was no assessment of data quality in the included studies.

### Data extraction

Data extraction for study characteristics and the four research questions was performed using a self-designed data extraction form in MS Excel. The extracted background information included authors, year of publication, country, the overarching objective of the study, sample size for VAS valuation, setting (university, clinics, etc.), information on study population (general population or specific disease groups), age, mode of administration, and if additional valuation methods had been used. Information relating to the four research questions was extracted, including purpose, health state definitions, perspective (own vs. described health state), design, and advantages and disadvantages of using a VAS identified by the authors of the articles. The latter were reported in the extraction form using quotes from the individual articles.

A subset of the articles deemed eligible for analysis was initially read independently by two reviewers (M.Å. and F.S.T.) who compared the findings to test the tabulation procedure. The majority of the articles were then read and tabulated by a third reviewer (Z.M.T.L.). Data extraction was continuously discussed among these reviewers and any disagreement was discussed and resolved among all authors.

The findings of the included studies were summarized and presented descriptively using frequencies and proportions of different categories in relation to the research questions. Articles where the VAS has been used in the context of economic evaluation were described separately, as this use has been subject to previous discussion [1, 3].

## Results

### Characteristics of included articles

**Fig. 1:**
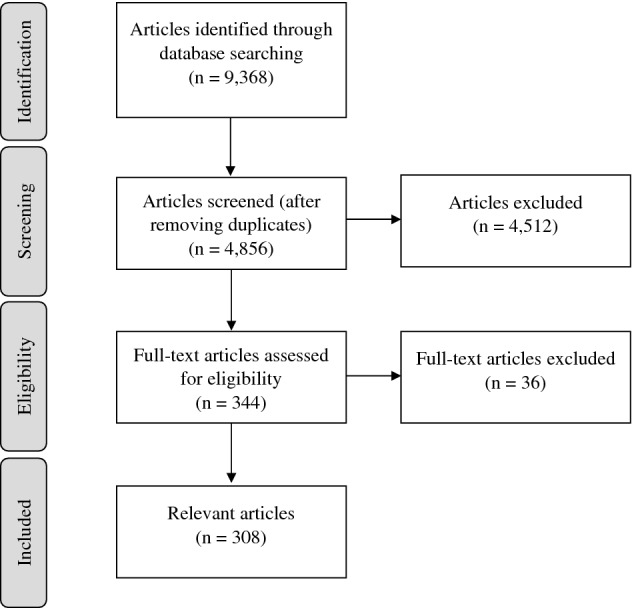
PRISMA flowchart illustrating selection of articles

After removing duplicates, the database search yielded 4856 articles (Fig. 1). Out of these, 344 articles were included as relevant and read in full text. Of these, 36 articles were excluded, yielding a total of 308 articles to be tabulated (Online Resource Table S2). Characteristics of the included articles are displayed in Table 1. The included articles were published between 1991 and 2021 (Online Resource Fig. S1).

**Table 1:** Study characteristics of the included articles (n = 308)

Study characteristics	n	%
Age groups		
Adults; ≥ 18 years	266	86.4
Children; < 18 years	8	2.6
All ages	26	8.4
Not reported	8	2.6
Sample sizes		
≤ 100	81	26.3
101–499	134	43.5
≥ 500	90	29.2
Not reported	3	1.0
Study settings		
Community^a^	109	35.4
Health care facilities^b^	96	31.2
Telephone and web	28	9.1
Schools and universities	15	4.9
Multiple settings	42	13.6
Not reported	18	5.8
Study population		
Patients	120	39.0
General population	114	37.0
Patients and others^c^	32	10.4
Children, parents and others^d^	26	8.4
Other populations^e^	14	4.5
Not reported	2	0.6
Mode of administration		
Interview		
Face-to-face interview	136	44.2
Computer-assisted interview	13	4.2
Web-based interview	3	1.0
Telephone interview	4	1.3
Self-administered questionnaire		
Questionnaire (without specification)	74	24.0
Web-based questionnaire	31	10.1
Mailed questionnaire	19	6.2
Computer-based questionnaire	12	3.9
Panel session	1	0.3
Secondary data	1	0.3
Mixed modes of administration	9	2.9
Not reported	5	1.6
VAS combined with other valuation methods		
No other methods	96	31.2
One other method		
TTO	89	28.9
SG	39	12.7
DCE	3	1.0
Ranking	2	0.6
WTO	1	0.3
Thurstone	1	0.3
More than one other method	77	25.0

*DCE* discrete choice experiment, *SG* standard gamble, *TTO* time trade-off, *VAS* visual analogue scale, *WTO* waiting trade-off

^a^Included communities, homes and offices

^b^Included hospitals, clinics, medical centres, primary care centres, nursing homes and laboratories

^c^Included general population, health care personnel, health individuals, partners, proxies, caregivers, family members, and students

^d^Included healthcare personnel, healthy individuals, general population, teachers and students

### Overall purposes of using a VAS for health state valuation

In 83% of the articles (n = 255) the main stated purpose for using a VAS was to value health states (Table 2). The remaining articles did use a VAS for health state valuation, but this was not expressed as the main purpose by the authors. Some articles also had an additional purpose, including rating disease severity (n = 3), comparing treatments (n = 2) or disease conditions (n = 1), and rating pain intensity (n = 1). Some articles (n = 20) used a VAS to value health states for comparison among respondents, disease conditions, treatments or with other valuation methods. Development of a country-specific value set was stated as the main purpose in five articles. In another 14 articles, the stated purpose of using a VAS was for economic evaluation.

**Table 2:** Reported purposes of using a VAS in the included studies (n = 308)

Stated purposes of using a VAS	n	%
For valuation of health states		
To value health states	255	82.8
To value health states and to rate disease severity	3	1.0
To value health states and to compare treatments	2	0.6
To value health states and to compare disease conditions	1	0.3
To value health states and to rate pain intensity	1	0.3
For economic evaluation	14	4.5
For comparison		
To compare respondents	8	2.6
To compare with other methods	6	1.9
To compare disease conditions	3	1.0
To compare with other methods and compare respondents	2	0.6
To compare treatments	1	0.3
For developing a country-specific value set	5	1.6
Other purposes		
To introduce valuation tasks	2	0.6
To present an anchoring method	1	0.3
To rate the importance of QoL aspects	1	0.3
To select health states for valuation studies	1	0.3
To assess scale recalibration of upper VAS anchor	1	0.3
To test validity of a utility assessment tool	1	0.3

*QoL* quality of life, *VAS* visual analogue scale

### Description of articles where a VAS was used for economic evaluation

For economic evaluations (Table 3), a VAS was used as a stand-alone method (n = 12) or in combination with another valuation method such as TTO (n = 2). Thirteen studies applied a VAS as a measure for calculating quality-adjusted life years (QALYs)—by using the VAS score as a utility value for the health states—and measuring cost-effectiveness of interventions. The remaining study used a VAS to rate health states and elicit willingness-to-pay values.

**Table 3:** Studies that used a VAS for economic evaluation (n = 14)

References	Country	Utility measures	Measure of effectiveness or cost-effectiveness
Arakawa et al. [13]	Japan	VAS	Cost per QALY
Bobinac et al. [71]	Netherlands	EQ-5D and EQ VAS	WTP per QALY
Brouwers et al. [61]	Netherlands	EQ-5D and EQ VAS	Cost per QALY
Bulthuis et al. [72]	Netherlands	SF-6D and VAS	Cost per QALY
Cheng et al. [62]	US	VAS, TTO and HUI	Cost per QALY
Johnson et al. [73]	US	VAS	Cost per QALY
Seidl et al. [14]	Germany	EQ-5D and VAS (for sensitivity analysis)	Cost per QALY
Sekigami et al. [74]	US	VAS	Cost per QALY
Sheckter et al. [75]	US	VAS	Cost per QALY
Shih et al. [76]	Singapore	VAS	Cost per QALY
Takura et al. [77]	Japan	EQ-5D and VAS (as an effectiveness measure)	Cost per QALY
Wong et al. [78]	US and Canada	VAS	QALY
Yu et al. [39]	US	VAS	Cost per QALY
Jia et al. [57]	Netherlands	RAND-36 and VAS	Cost per QALY

*EQ VAS* EuroQol visual analogue scale, *HUI* Health Utilities Index, *QALY* quality-adjusted life-year, *RAND-36* rand 36-item health survey, *SF-6D* six-dimensional health state short form, *TTO* time trade-off, *VAS* visual analogue scale, *WTP* willing to pay

In one study, the authors justified the use of a VAS for measuring utilities in referencing Drummond et al. [3], stating that preferences for chronic health states can be measured by using a rating scale [13]. They also claimed that the EQ-5D descriptive system could not detect utility values for certain health dimensions related to endometriosis [13]. One study used a VAS in a sensitivity analysis, as it is a direct method for self-rating health [14].

### Definitions of health states valued

In a majority of studies (61%), a disease-specific health state or patients’ current health state were valued (Table 4), followed by EQ-5D health states (n = 67). Some studies included additional states, such as dead/death and unconscious (n = 7), and dead/death (n = 3). In some studies, a VAS was used to value current health states in the general population (n = 22). Some studies included both current health states from the general population and disease-specific health states (n = 9).

**Table 4:** Definition of health states valued with a VAS in the included articles (n = 308)

Definitions of health states valued with a VAS	n	%
Disease states	188	61.0
EQ-5D states		
EQ-5D states only	67	21.8
EQ-5D states, dead/death and unconscious	7	2.3
EQ-5D states and dead/death	3	1.0
EQ-5D and disease states	3	1.0
EQ-5D states and current health states of general population	1	0.3
EQ-5D and SF-6D states	2	0.6
Current health states		
Current health states of general population only	22	7.1
Current health states of general population and disease states	9	2.9
Other states		
AQoL-8D states	1	0.3
HUI2 states	1	0.3
HUI3 states	1	0.3
QOLIBRI-OS states	1	0.3
SF-6D states	1	0.3
15D states	1	0.3

*AQoL-8D* assessment of quality of life eight dimension, *HUI2* Health Utilities Index Mark 2, *HUI3* Health Utilities Index Mark 2, *QOLIBRI-OS* quality of life after brain injury overall scale, *SF-6D* six-dimensional health state short form, *VAS* visual analogue scale

**Fig. 2:**
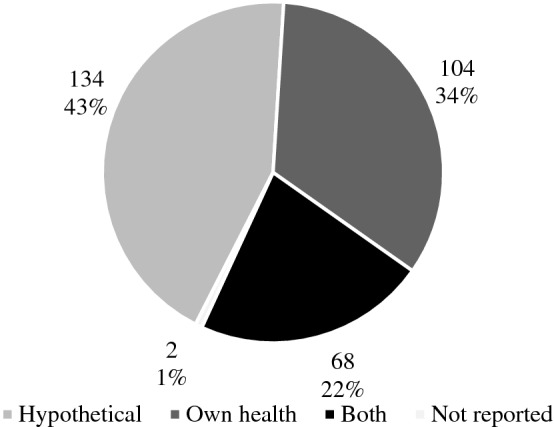
Number and proportion of studies using hypothetical, own health or both perspectives using the VAS method for health state valuation

The two most commonly used perspectives when valuing health states with a VAS were hypothetical (44%) and own health (34%) (Fig. 2). In 22% of the studies, both hypothetical health states and own health were valued.

### Designs of the VAS

In about half of the studies it was mentioned that the EQ VAS was used (Online Resource Table S3), in 10% of the studies the vertical EQ VAS with 10, 20 or 50 cm in length, while in 37% the design was not explicitly described (Online Resource Table S3). In the other half of the studies, an unspecified VAS was used, where 32% provided no further description of the design. Apart from this, an erectile function (EF) visual analogue scale (EF-VAS) was used in one study which applied both disease-specific and generic VAS scales in valuing EF-related health states.

### Anchoring

Seventy percent of the included studies used 0–100 as anchors for the VAS; in 16 studies 0–1 was used, and in five 0–10 (Online Resource Table S4). Two studies included negative values for health states worse than death as the lower anchor, and 100 or 10 as the upper anchor, respectively. The most commonly (38%) used anchors were 0 for the worst imaginable health state and 100 for the best imaginable health state, 0 for dead/death and 100 for perfect health (16%), and dead/death to perfect health, without numbers (6%). The description of the lower and upper anchors varied to quite a large degree, see Online Resource Table S4. In contrast, one study reported the use of 0 for no problems and 100 for the worst imaginable problems.

### Reported advantages and disadvantages of using a VAS

A majority of the included studies (86%) did not report advantages or disadvantages of using the VAS, mainly because it was not the focus of the studies. From the studies where the authors reflected their own opinions, the information was extracted and presented in Online Resource Tables S5 (advantages) and S6 (disadvantages).

Administration of the VAS questionnaire was described as simple, easy to understand, taking shorter time and being less costly to conduct (Table S5) [15–20]. Using a VAS was stated as a practical approach to valuing health states in the general population, including children, and certain patient populations [18, 21]. When comparing different formats of the VAS, scoring was suggested to be a potentially more intuitive approach than drawing lines [22].

Compared to SG and TTO, the VAS was perceived as an easier and more practical approach [17, 23–26]. Consequently, using a VAS was associated with lower respondent burden and administration costs, and fewer measurement errors [27]. The VAS was seen as more sensitive or discriminatory to symptoms and more culturally acceptable [25, 28]. The VAS was observed to have a better model fit and a similar predictive ability compared to the TTO [25, 29].

When using the VAS in research practice, it was described as a feasible and acceptable valuation method due to its simplicity, reliability, validity and practicality in valuing health states as a stand-alone tool or in combination with other methods [16, 17, 19, 24, 30–34]. The VAS was sensitive and could capture the variability of changes in health states [16, 18, 35]. It was also seen to be able to capture health information which might not be reflected in pre-defined health profiles and to serve as a proxy for effects otherwise missed [36–38]. In addition, the VAS was reported to have a good predictive ability, as patients with higher initial VAS scores were observed to require fewer healthcare visits [38]. It was also described as a simple and effective tool for economic evaluations and seen as useful for decision-making in everyday practice [39, 40].

In terms of disadvantages, some authors preferred other valuation methods, such as SG and TTO, over a VAS (Online Resource Table S6). The most common criticism was that the VAS does not incorporate a risk or trade-off and therefore does not measure utilities or lacks the conceptual richness when capturing preferences [17, 23, 25, 26, 41–45]. TTO was seen as likely to discriminate better between health states than a VAS [27, 46]. The VAS and SG were reported to have similar difficulty levels in usage [47]. Both measures were seen as insensitive to improvements in patients with breathing-related symptoms [42].

VAS was claimed not to be able to measure utilities or preferences on a cardinal scale [14, 48]. The VAS might produce systematically lower scores than other instruments due to the lack of a trade-off property [49]. Its validity was questionable in valuation of certain health conditions, such as pelvic floor disorders, urinary incontinence and faecal incontinence [50–52]. The VAS was seen as prone to bias and had low correlation with other generic and condition-specific HRQoL instruments [48, 52]. Finally, the VAS was criticized for not adding much explanatory power to the models that were used to explain TTO values [53].

## Discussion

This study set out to review how the VAS has been used for health state valuation to date. We included a total of 308 articles published between 1991 and 2021 from 40 countries in six continents. A rapid rise in relevant publications was observed in 1998 after the introduction of EuroQol health state valuation by Dolan [54], and in 2005 after the EQ-5D was included by the National Institute for Health and Care Excellence (NICE) as a preferred instrument for technology appraisal [55].

Most of the reviewed studies were community-based or healthcare facility-based with varying sample sizes of participants aged 18 years and above. Participants under 18 years were included in some studies among which the youngest participants were aged 5 years [56, 57]. A previous study has shown VAS to be reliable from 5 years [58].

In most studies, a VAS was used together with one or more other valuation methods. One of the main reasons not to use the VAS alone is that it can be prone to measurement bias [5]. Despite this, the VAS has been shown to have its own advantages as well [1, 3, 59]. Taking the generic EQ-5D instrument as an example, the EQ VAS provides complementary information to the EQ-5D profile, as it reflects the overall health status and probably captures other aspects of health not included in the EQ-5D descriptive system [60].

Regarding modes of administration, telephone, web-based or postal studies have become popular since 2001. This trend can be expected to grow, particularly in the post-COVID era in which unnecessary physical interactions may be reduced. The use of VAS also requires minimum explanation to respondents, and self-administration is generally easier when valuing health states with the VAS compared to SG or TTO [5].

Despite the ongoing discussions on whether VAS valuations are appropriate for use in cost-utility analysis, we identified 13 studies which applied a VAS for this purpose, mostly as a stand-alone method. In fact, the use of a VAS is accepted as the simplest approach to measuring preferences for both chronic and temporary health states [3]. It is also seen as a direct measure of self-rated health and as able to provide additional information by measuring health on a single dimension [1].

The main criticism regarding the use of VAS in economic evaluation is that it does not measure utility under uncertainty [1, 3]. However, Parkin and Devlin [59] argue that QALYs do not need to be based on utility theory as the use of QALYs in economic evaluation is primarily to inform the allocation of limited resources for improvements of health rather than utility [59]. They argue that using the VAS involves both choice and trade-off across sets of health states, and that other methods also suffer from biases or concerns regarding generating reliable preferences [59]. Although our scoping review did not yield any further theoretical arguments, it shows that the VAS has been used empirically in a number of economic evaluation studies since 2000. In these studies, many in a clinical context, an important attribute of VAS as a method easier to use than other valuation methods has been demonstrated. This was particularly exemplified by two of the reviewed economic evaluations which employed power functions to transform VAS to TTO values which were comparable to TTO elicited values [61, 62], illustrating the practicality of VAS valuation and the possibility to generate values equivalent to TTO.

For the health states valued using VAS, the most common ones were disease-specific or related to the current health status of respondents, indicating the usefulness of a VAS for decisions in everyday clinical practice [63]. In addition, as the EQ VAS is part of the EQ-5D instrument it was not surprising that the VAS was used to value EQ-5D health states, alone or in combination with other valuation methods.

There can be variations in the design when applying the VAS method. The line can be vertical or horizonal, vary in length, and be with or without intervals marked for different numbers [1]. The response on the VAS is also indicated differently, such as drawing a line to indicate the position of a health state [1, 2, 60] or placing cards to describe different levels of health states [64]. Confirming earlier observations by Brazier et al. [1], our review showed a large variation in the designs of a VAS. Such heterogeneity in VAS designs could have implications for the field by affecting comparability and uniformity of studies. An early study comparing horizontal and vertical VAS reported very high correlation [65]. In contrast, a relatively recent study comparing a patient global assessment VAS (10 cm, horizontal) and the EQ VAS reported moderate correlation with poor concordance [66]. A systematic review on design differences in paper-based and electronic VAS reported equivalence of results despite differences in scale length and format [67]. Use of standardized and clear descriptions of VAS designs used in specific studies could improve uniformity across studies and in turn facilitate comparisons of results. The diversity in design can also be seen in other valuation methods. For example, regarding the TTO, both open-ended and iteration based TTO tasks have been used [68]. There has also been a development of the TTO task over time, for example regarding health states worse than dead [69]. Implications of these variations, as well as interview effects and how cognitively challenging the task is, need to be addressed further for all valuation methods.

There are several ways of anchoring the VAS, for example at 1 (‘full health’) and 0 (‘dead’). Health states valued as worse than ‘dead’ are consequently assigned negative values. However, it has been argued that anchoring 0 at ‘dead’ is not a theoretical requirement for health status measurement or for cost-utility analysis [70]. While the terms ‘dead’ and ‘death’ are sometimes used interchangeably, the choice of term may matter in valuation studies, as ‘dead’ always refers to a state, but ‘death’ can mean either a state or an event [70]. Despite the variations of the anchors used, the most common ones were 0 for the worst imaginable health state and 100 for the best imaginable health state. Some studies also used the lower anchor of 0 for dead/death and negative values for the states worse than dead/death. Amongst these, a majority used the term ‘death’ for the lower anchor while only a few used the term ‘dead’. This seems to be opposed to what was suggested by OHE, seeing ‘dead’ as a more representative term for a state than ‘death’ which could be ambiguous [70]. However, the phrasing is not always internally consistent, as some authors used the terms ‘dead’ and ‘death’ interchangeably in the same manuscript, and the same authors sometimes used different terms in other publications. This could be due to different journals’ requirements or changes in the field over time. Harmonization of the endpoints of the VAS would enable the comparison of results. Furthermore, conducting studies using qualitative methods to explore how the phrasing of different endpoints are perceived among participants is recommended.

Among the articles that reported advantages and disadvantages of using a VAS, more advantages were mentioned, which could also be due to our selection criteria (focusing on articles that used a VAS). Common advantages stated were its simplicity, reliability, validity, and practicality in health state valuation, which consequently lead to feasibility and acceptability in its application. The applicability of VAS in economic evaluations was also acknowledged by some authors.

There were also criticisms regarding using a VAS as a utility measure, based on methodological considerations. These include poor theoretical foundations and no risk or trade-off property, although arguments against these criticisms have been put forward by Parkin and Devlin [59] as discussed above. Some authors also questioned its validity in valuing certain health conditions and the explanatory power of resulting models.

### Strengths and limitations

The main strength of our study is its comprehensive and systematic coverage of the literature. At least two reviewers were engaged in screening and selection of the articles and extraction of data in order to minimize bias throughout the process.

A limitation is that no quality assessment of the reviewed articles was conducted. However, the aim was to describe how the VAS had been used for health state valuations in the published literature and not to judge the obtained values from these studies. Our review focused on applications of the VAS, which means that many of the included articles did not systematically report observed advantages or disadvantages of using a VAS, and more theoretical arguments are likely to be found in other types of literature. Another shortcoming is that there may be other articles where the VAS has been used in economic evaluations which we did not include as our search strategies and selection criteria were primarily targeted towards valuation studies, following from our aim to describe how VAS has been used for health state valuation.

## Conclusions

This scoping review shows that the VAS is a common method for valuing various types of health states, both as a stand-alone method and in combination with other valuation methods such as the SG and TTO. In addition to the purpose of health state valuation, the VAS has been applied in economic evaluations, which can provide valuable input into ongoing discussions in this area. Harmonization of the design of the VAS when used for health state valuation and future qualitative studies to explore how participants perceive different endpoints of the VAS are recommended. Furthermore, more research on the role and consequences of using the VAS in economic evaluations is warranted.

## Supplementary Information

Below is the link to the electronic supplementary material.

Supplementary file 1 (PDF 939 kb)

## Data Availability

Not applicable.
