# Supplementary material for: Use of the visual analogue scale for health state valuation: a scoping review
Source: Qual Life Res. 2023 Apr 7;32(10):2719–29. doi: 10.1007/s11136-023-03411-3 (PMC10474194; doi:10.1007/s11136-023-03411-3)
Supplement: Supplementary file 1 — (PDF 939 kb) [file 11136_2023_3411_MOESM1_ESM.pdf]

SUPPLEMENTARY MATERIALS

Title: Use of the Visual Analogue Scale for health state valuation: A scoping review

Journal: Quality of Life Research

AUTHORS: Mimmi Åström[1,2,3*], Zin Min Thet Lwin[1*], Fitsum Sebsibe Teni[1],

Kristina Burström[1,2], Jenny Berg[1]

1. Health Outcomes and Economic Evaluation Research Group, Stockholm Centre for Healthcare Ethics, Department of Learning, Informatics, Management and Ethics, Karolinska Institutet, Stockholm, Sweden.
2. Equity and Health Policy Research Group, Department of Global Public Health, Karolinska Institutet, Stockholm, Sweden.
3. Centre for Health Economics, Informatics and Health Services Research, Stockholm Health Care Services, Region Stockholm, Sweden

*The first two authors contributed equally to this work

Mimmi Åström, PhD; ORCID: 0000-0002-6711-5262

Zin Min Thet Lwin, MSc; ORCID: 0000-0003-4305-6728

Fitsum Sebsibe Teni, MSc, MPH; ORCID: 0000-0002-6182-499X

Kristina Burström, PhD, Associate professor; ORCID: 0000-0001-9996-4317

Jenny Berg, PhD, ORCID: 0000-0002-5994-6816

Corresponding author:

Mimmi Åström

Tel: +46 70 284 71 62, e-mail: mimmi.astrom@ki.se

Contents

Table S1: Search Strategies for the Databases Medline (OVID), Web of Science (Clarivate) and PsycInfo (OVID) 4

Table S2: Reference List to Included Articles (n = 308) 7

Table S3: Designs of the VAS used in the included studies (n = 308) 28

Table S4: Types of anchoring for the VAS used in the included studies (n = 308) 30

Table S5: Reported advantages of using a VAS in the included studies 33

Table S6: Reported disadvantages of using a VAS in the included studies 37

Figure S1: Number of Included Studies by Publication Year (n = 308) 40

Figure S2: Countries Where the Included Studies Were Conducted (n = 308) 40

Despite yearly fluctuations, the number of articles published per year increased gradually from two in 1991 to 11 in 2021. The studies used data from 40 countries from the six continents Africa, Asia, Europe, Oceania, North and South America (Fig. S2 in Supplementary Material). A majority of the studies were from the United States (US) (n=83), the United Kingdom (UK) (n=55), the Netherlands (n=46), Canada (n=35), Germany (n=17), South Korea (n=13) and Spain (n=12).

For each characteristic, there were articles that did not report the specific information. In most studies (86%, n=266), the participants were aged 18 years and above. The sample size varied: 26% had a sample size of less than 100 participants and in 29% of the studies the sample size was greater than 500 participants.

The most common study settings were community-based (35%, n=109), healthcare facility-based (31%, n=96), followed by telephone or web-based (9%, n=28), and schools or universities (5%, n=15).

Specific patient populations were included in 120 (39%) of the articles and 114 (37%) sampled from the general population. The remaining articles included a mix of these populations and, e.g., health care personnel, students, teachers, parents and children.

The most common mode of administration for the VAS valuation was face-to-face interview (44%, n=136), followed by use of a questionnaire (24%, n=74). In addition, computer-assisted interview (n=13), web-based interview (n=3), telephone interview (n=4), web-based questionnaire (n=31), mailed questionnaire (n=19), computer-based questionnaire (n=12) have been employed. Secondary data was used in one study, panel session was used in another, and in eight other studies more than one method was used.

A VAS was used as a stand-alone valuation method in 96 articles (31%), whereas in 135 articles (44%) another valuation method, for example, TTO, SG, DCE or Ranking was used together with a VAS.

Table S1: Search Strategies for the Databases Medline (OVID), Web of Science (Clarivate) and PsycInfo (OVID)

No.	Search in Medline (OVID)
1	Visual Analog Scale/
2	((visual analog* scale or VAS or EQ-5D) adj5 (adjust* or evaluat* or estimat* or experience* or rate* or rating or utilit* or valu*)).ti,ab,kf.
3	or/1-2
4	Health Status/
5	Quality of life/
6	Quality-Adjusted Life Years/
7	((health stat* or quality) adj5 (adjust* or evaluat* or estimat* or experience* or rate* or rating or utilit* or valu*)).ti,ab,kf.
8	((HRQOL or QALY) adj5 (adjust* or evaluat* or estimat* or experience* or rate* or rating or utilit* or valu*)).ti,ab,kf.
9	or/4-8
10	3 and 9
11	limit 10 to english language
12	review.pt.
13	systematic review.pt.
14	review*.ti.
15	or/12-14
16	11 not 15
17	11 not 16

exp/ = exploded MeSH term

/ = non exploded MeSH term

.ti,ab,kf. = title, abstract and author keywords

adjx = adjacent within x words, regardless of order

* = truncation of word for alternate endings

No.	Search in Web of Science (Clarivate)
1	TOPIC: ((("visual analog* scale" or VAS or EQ-5D) NEAR/5 (adjust* or evaluat* or estimat* or experience* or rate* or rating or utilit* or valu*)))<br>AND
2	TOPIC: ((("health stat*" or quality) NEAR/5 (adjust* or evaluat* or estimat* or experience* or rate* or rating or utilit* or valu*))) OR TOPIC: (((HRQOL or QALY) NEAR/5 (adjust* or evaluat* or estimat* or experience* or rate* or rating or utilit* or valu*)))
3	Refined by: LANGUAGES: (ENGLISH) AND DOCUMENT TYPES: (ARTICLE OR REVIEW OR OTHER OR EARLY ACCESS OR CLINICAL TRIAL OR CASE REPORT)

TS/Topic = title, abstract, author keywords and Keywords Plus
NEAR/x = adjacent within x words, regardless of order
* = truncation of word for alternate endings

No.	Search in PsycInfo (OVID)
1	((Visual analog* scale or VAS or EQ-5D) adj5 (adjust* or evaluat* or estimat* or experience* or rate* or rating or utilit* or valu*)).ti,ab,id.
2	health status/
3	exp quality of life/
4	"quality of life measures"/
5	((health stat* or quality) adj5 (adjust* or evaluat* or estimat* or experience* or rate* or rating or utilit* or valu*)).ti,ab,id.
6	((HRQOL or QALY) adj5 (adjust* or evaluat* or estimat* or experience* or rate* or rating or utilit* or valu*)).ti,ab,id.
7	or/2-6
8	1 and 7
9	limit 8 to english language
10	review*.ti.
11	9 and 10
12	9 not 11

exp/ = exploded controlled term

/ = non exploded controlled term

.ti,ab,id. = title, abstract and author keywords

adjx = adjacent within x words, regardless of order

* = truncation of word for alternate endings

Table S2: Reference List to Included Articles (n = 308)

Abt NB, Quatela O, Heiser A, et al. Association of hair loss with health utility measurements before and after hair transplant surgery in men and women. *JAMA Facial Plast Surg*. 2018;20(6):495-500. doi:10.1001/jamafacial.2018.1052

Aldebeyan S, Sinno H, Makhdom A, Ouellet JA, Saran N. Impact of living with scoliosis: A utility outcome score assessment. *Spine*. 2017;42(2):E93-E97. doi:10.1097/BRS.0000000000001708

Aldihan K, Alnasyan A, Albassam A, Alghonaim Y, Aldekhayel S. Comparing the Health Burden of Living with Nasal Deformity in Actual Patients and Healthy Individuals: A Utility Outcomes Score Assessment. *Ann Plast Surg*. 2019;83(4):381-383. doi:10.1097/SAP.0000000000002065

Alshiek J, Garcia B, Minassian VA, et al. Vaginal Energy Based Devices - AUGS Clinical Consensus Statement. *Female Pelvic Med Reconstr Surg.* 2020;26(5):287-298. doi:10.1097/SPV

Andrade MV, Noronha K, Kind P, De Barros Reis C, De Carvalho LR. Logical inconsistencies in 3 preference elicitation methods for EQ-5D health states. *Med Decis Mak*. 2016;36(2):242-252. doi:10.1177/0272989X15601047

Arakawa I, Momoeda M, Osuga Y, Ota I, Koga K. Cost-effectiveness of the recommended medical intervention for the treatment of dysmenorrhea and endometriosis in Japan. *Cost Eff Resour Alloc*. 2018;16(1):1-10. doi:10.1186/s12962-018-0097-8

Arons AMM, Krabbe PFM, Schölzel-Dorenbos CJM, Van Der Wilt GJ, Olde Rikkert MGM. Thurstone scaling revealed systematic health-state valuation differences between patients with dementia and proxies. *J Clin Epidemiol*. 2012;65(8):897-905. doi:10.1016/j.jclinepi.2012.01.018

Arons AMM, Krabbe PFM, Van Der Wilt GJ, Olde Rikkert MGM, Adang EMM. Visual analogue scales: Scale recalibration by patients with dementia and their proxies. *Qual Life Res*. 2013;22(5):979-986. doi:10.1007/s11136-012-0226-3

Åström M. Health State Utilities for Patients Current Health from Bipolar Type I Disorder. *J Ment Health Policy Econ*. 2018;21(1).

Åström M. Measurement of the quality of life during different clinical phases of breast cancer. *J Formos Med Assoc*. 1999;98(4):254-260.

Åström M. Can health utility measures be used in lupus research? A comparative validation and reliability study of 4 utility indices. *J Rheumatol*. 1999;26(6).

Attema AE, Brouwer WBF, Prades JLP. Peer effects in health valuation: The relation between rating of contemporaries' health and own health. *Health Qual Life Outcomes*. 2018;16(1):1-9. doi:10.1186/s12955-018-0978-8

Augestad LA, Rand-Hendriksen K, Stavem K, Kristiansen IS. Time trade-off and attitudes toward euthanasia: Implications of using "death" as an anchor in health state valuation. *Qual Life Res*. 2013;22(4):705-714. doi:10.1007/s11136-012-0192-9

Augustovski FA, Irazola VE, Velazquez AP, Gibbons L, Craig BM. Argentine valuation of the EQ-5D health states. *Value Heal*. 2009;12(4):587-596. doi:10.1111/j.1524-4733.2008.00468.x

Auvin S, Damera V, Martin M, Holland R, Simontacchi K, Saich A. The impact of seizure frequency on quality of life in patients with Lennox-Gastaut syndrome or Dravet syndrome.

Epilepsy Behav. 2021;123. doi:10.1016/j.yebeh.2021.108239

Badia Llach X, Monserrat S, Roset M, Herdman M. Feasibility, validity and test-retest reliability of scaling methods for health states: The visual analogue scale and the time trade-off. *Qual Life Res*. 1999;8(4):303-310. doi:10.1023/A:1008952423122

Badia X, Herdman M, Kind P. The influence of ill-health experience on the valuation of health. *Pharmacoeconomics*. 1998;13(6):687-696. doi:10.2165/00019053-199813060-00005

Badia X, Roset M, Herdman M. Inconsistent responses in three preference-elicitation methods for health states. *Soc Sci Med*. 1999;49(7):943-950. doi:10.1016/S0277-9536(99)00182-3

Bakhshandeh H, Naeini KH, Zeraati H, et al. Health state valuation in Iran: An exercise on Cardiovascular diseases using visual analogue scale method. *Iran J Public Health*. 2009;38(4):46-55.

Ballinger R, Macey J, Lloyd A, et al. Measurement of Utilities Associated with Parenteral Support Requirement in Patients with Short Bowel Syndrome and Intestinal Failure. *Clin Ther*. 2018;40(11):1878-1893.e1. doi:10.1016/j.clinthera.2018.09.009

Barbist MT, Renn D, Noisternig B, Rumpold G, Höfer S. How do medical students value health on the EQ-5D? Evaluation of hypothetical health states compared to the general population. *Health Qual Life Outcomes*. 2008;6:111. doi:10.1186/1477-7525-6-111

Bhatnagar V, Frosch DL, Tally SR, et al. Evaluation of an Internet-based disease trajectory decision tool for prostate cancer screening. *Value Heal*. 2009;12(1):101-108. doi:10.1111/j.1524-4733.2008.00407.x

Bijlenga D, Birnie E, Bonsel GJ. Feasibility, reliability, and validity of three health-state valuation methods using multiple-outcome vignettes on moderate-risk pregnancy at term. *Value Heal*. 2009;12(5):821-827. doi:10.1111/j.1524-4733.2009.00503.x

Bijlenga D, Birnie E, Mol BWJ, Bonsel GJ. Obstetrical outcome valuations by patients, professionals, and laypersons: Differences within and between groups using three valuation methods. *BMC Pregnancy Childbirth*. 2011;11. doi:10.1186/1471-2393-11-93

Bobinac A, Van Exel NJA, Rutten FFH, Brouwer WBF. Willingness to pay for a quality-adjusted life-year: The individual perspective. *Value Heal*. 2010;13(8):1046-1055. doi:10.1111/j.1524-4733.2010.00781.x

Botes R, Vermeulen KM, Ranchor A V., Buskens E. Functional health state description and valuation by people aged 65 and over: A pilot study. *BMC Geriatr*. 2018;18(1):1-8. doi:10.1186/s12877-018-0711-9

Brazier J, Usherwood T, Harper R, Thomas K. Deriving a preference-based single index from the UK SF-36 Health Survey. *J Clin Epidemiol*. 1998;51(11):1115-1128. doi:10.1016/S0895-4356(98)00103-6

Brouwers RWM, Van Der Poort EKJ, Kemps HMC, Van Den Akker-Van Marle ME, Kraal JJ. Cost-effectiveness of Cardiac Telerehabilitation with Relapse Prevention for the Treatment of Patients with Coronary Artery Disease in the Netherlands. JAMA Netw Open. 2021;4(12):2-15. doi:10.1001/jamanetworkopen.2021.36652

Bulthuis Y, Mohammad S, Braakman-Jansen LMA, Drossaers-Bakker KW, Van De Laar MAFJ. Cost-effectiveness of intensive exercise therapy directly following hospital discharge in patients with

arthritis: Results of a randomized controlled clinical trial. *Arthritis Care Res*. 2008;59(2):247-254. doi:10.1002/art.23332

Burström K, Sun S, Gerdtham UG, et al. Swedish experience-based value sets for EQ-5D health states. *Qual Life Res*. 2014;23(2):431-442. doi:10.1007/s11136-013-0496-4

Burström K, Teni FS, Gerdtham UG, et al. Experience-Based Swedish TTO and VAS Value Sets for EQ-5D-5L Health States. *Pharmacoeconomics*. 2020;38(8):839-856. doi:10.1007/s40273-020-00905-7

Butt T, Dunbar HMP, Morris S, Orr S, Rubin GS. Patient and public preferences for health states associated with AMD. *Optom Vis Sci*. 2013;90(8):855-860. doi:10.1097/OPX.0b013e3182962318

Byun S, Hong P, Bezuhly M. Public perception of the burden of microtia. *J Craniofac Surg*. 2016;27(7):1665-1669. doi:10.1097/SCS.0000000000002900

Campolina AG, Bortoluzzo AB, Ferraz MB, Ciconelli RM. Validity of the SF-6D index in Brazilian patients with rheumatoid arthritis. *Clin Exp Rheumatol*. 2009;27(2):237-245.

Castejón N, Cappelleri JC, Cuervo J, et al. Social preferences for health states associated with acute myeloid leukemia for patients undergoing treatment in the United Kingdom. *Health Qual Life Outcomes*. 2018;16(1):1-9. doi:10.1186/s12955-018-0897-8

Chancellor J, Aballéa S, Lawrence A, et al. Preferences of patients with diabetes mellitus for inhaled versus injectable insulin regimens. *Pharmacoeconomics*. 2008;26(3):217-234. doi:10.2165/00019053-200826030-00005

Chang WT, Collins ED, Kerrigan CL. An internet-based utility assessment of breast hypertrophy. *J Am Soc Plast Surg*. 2000;108(2):370-377.

Chapman BP, Franks P, Duberstein PR, Jerant A. Differences between individual and societal health state valuations: Any link with personality? *Med Care*. 2009;47(8):902-907. doi:10.1097/MLR.0b013e3181a8112e

Chatroux L, Darney BG, Skye M, Caughey AB, Rodriguez MI. Examining the effect of unintended pregnancy on health utility using four measures. Contraception. 2021;104(4):344-349. doi:10.1016/j.contraception.2021.04.019

Cheng AK, Rubin HR, Powe NR, Mellon NK, Francis HW, Niparko JK. Cost-utility analysis of the cochlear implant in children. *J Am Med Assoc*. 2000;284(7):850-856. doi:10.1001/jama.284.7.850

Chiou CF, Weaver MR, Bell MA, Lee TA, Krieger JW. Development of the multi-attribute pediatric asthma health outcome measure (PAHOM). *Int J Qual Heal Care*. 2005;17(1):23-30. doi:10.1093/intqhc/mzh086

Chiou CF, Weisman M, Sherbourne CD, et al. Measuring preference weights for american college of rheumatology response criteria for patients with rheumatoid arthritis. *J Rheumatol*. 2005;32(12):2326-2329.

Cho S, Kim H, Kim SH, et al. Utility estimation of hypothetical chronic obstructive pulmonary disease health states by the general population and health professionals. *Health Qual Life Outcomes*. 2015;13(1):1-7. doi:10.1186/s12955-015-0228-2

Chou TC, Chiang SC, Ko Y. Health state utilities for metastatic breast cancer in Taiwan. *Breast*. 2020;51:57-64. doi:10.1016/j.breast.2020.03.001

Chuang LH, Kind P. The effect of health state selection on the valuation of EQ-5D. *Med Decis*

Mak. 2011;31(1):186-194. doi:10.1177/0272989X10371231

Clarke P, Gray A, Holman R. Estimating utility values for health states of type 2 diabetic patients using the EQ-5D (UKPDS 62). *Med Decis Mak*. 2002;22(4):340-349. doi:10.1177/027298902400448902

Cleemput I, Kind P, Kesteloot K. Re-scaling social preference data: Implications for modelling. *Eur J Heal Econ*. 2004;5(4):290-298. doi:10.1007/s10198-004-0242-5

Cleemput I. A social preference valuations set for EQ-5D health states in Flanders, Belgium. *Eur J Heal Econ*. 2010;11(2):205-213. doi:10.1007/s10198-009-0167-0

Craig BM, Busschbach JJV, Salomon JA. Scale Values for EQ-5D Health States. *Med Care*. 2009;47(6):634-641.

Craig BM, Busschbach JJV, Salomon JA. Keep it simple: Ranking health states yields values similar to cardinal measurement approaches. *J Clin Epidemiol*. 2009;62(3):296-305. doi:10.1016/j.jclinepi.2008.07.002

Craig BM, Ramachandran S. Relative risk of a shuffled deck: A generalizable logical consistency criterion for sample selection in health state valuation studies. *Health Econ*. 2006;15(8):835-848. doi:10.1002/hec.1108

Craig BM. The duration effect: a link between TTO and VAS values. *Health Econ*. 2009;23(1):1-7. doi:10.1002/hec.1356.THE

Daar DA, Shah A, Mirrer JT, Thanik V, Hacquebord J. Proximal Row Carpectomy versus Four-Corner Arthrodesis for the Treatment of Scapholunate Advanced Collapse/Scaphoid Nonunion Advanced Collapse Wrist: A Cost-Utility Analysis. *Plast Reconstr Surg*. 2019;143(5):1432-1445. doi:10.1097/PRS.0000000000005558

David C, Efanov JI, Borsuk DE. Utility Outcome Measures for the Treatment of Ameloblastomas during Childhood. Plast Reconstr Surg - Glob Open. 2021;9(1):e3311. doi:10.1097/gox.0000000000003311

De Boer AGEM, Stalmeier PFM, Sprangers MAG, et al. Transhiatal vs extended transthoracic resection in oesophageal carcinoma: Patients' utilities and treatment preferences. *Br J Cancer*. 2002;86(6):851-857. doi:10.1038/sj.bjc.6600203

De Wit GA, Busschbach JJV, De Charro FT. Sensitivity and perspective in the valuation of health status: Whose values count? *Health Econ*. 2000;9(2):109-126. doi:10.1002/(SICI)1099-1050(200003)9:2<109::AID-HEC503>3.0.CO;2-L

Demeulemeester F, De Letter M, Miatton M, Santens P. Quality of life in patients with PD and their caregiving spouses: A view from both sides. *Clin Neurol Neurosurg*. 2015;139:24-28. doi:10.1016/j.clineuro.2015.08.027

Devlin N, Hansen P, Herbison P, Macran S. A "new and improved" EQ-5D valuation questionnaire? Results from a pilot study. *Eur J Heal Econ*. 2005;6(1):73-82. doi:10.1007/s10198-004-0263-0

Devlin NJ, Hansen P, Kind P, Williams A. Logical inconsistencies in survey respondents' health state valuations - A methodological challenge for estimating social tariffs. *Health Econ*. 2003;12(7):529-544. doi:10.1002/hec.741

DeVore EK, Shrime MG, Wittenberg E, et al. The health utility of mild and severe dysphonia. *Laryngoscope*. 2020;130(5):1256-1262. doi:10.1002/lary.28216

Diaz-Redondo A, Rodríguez-Blázquez C, Ayala A, et al. EQ-5D rated by proxy in institutionalized older adults with dementia: Psychometric pros and cons. *Geriatr Gerontol Int*. 2014;14(2):346-353. doi:10.1111/ggi.12108

Dillman JR, Carlos RC, Smith EA, et al. Relationship of bowel MR imaging to health-related quality of life measures in newly diagnosed pediatric small bowel Crohn disease. *Radiology*. 2016;280(2):568-575. doi:10.1148/radiol.2016151727

Doctor JN, Zoellner LA, Feeny NC. Predictors of Health-Related Quality-of-Life Utilities Among Persons With Posttraumatic Stress Disorder. Psychiatr Serv. 2011;62(3). doi:10.1176/appi.ps.62.3.272

Dolan P, Roberts J. To what extent can we explain time trade-off values from other information about respondents? *Soc Sci Med*. 2002;54(6):919-929. doi:10.1016/S0277-9536(01)00066-1

Dolan P, Sutton M. Mapping visual analogue scale health state valuations onto standard gamble and time trade-off values. *Soc Sci Med*. 1997;44(10):1519-1530. doi:10.1016/S0277-9536(96)00271-7

Dolan P, Torgerson D, Kakarlapud TK. Health-related quality of life of Colles fracture patients. *Osteoporos Int*. 1999;9(3):196-199. doi:10.1007/s001980050136

Dolan P. Modelling valuations for health states: The effect of duration. *Health Policy*. 1996;38(3):189-203. doi:10.1016/0168-8510(96)00853-6

Doyle S, Lloyd A, Walker M. Health state utility scores in advanced non-small cell lung cancer. *Lung Cancer*. 2008;62(3):374-380. doi:10.1016/j.lungcan.2008.03.019

Ebrahim S, Parshuram C. Comparison of utility scores from the Visual Analog Scale and Health Utilities Index 3 in children following pediatric intensive care unit admission. *J Child Heal Care*. 2015;19(1):53-62. doi:10.1177/1367493513496909

Edelaar-Peeters Y, Stiggelbout AM. Anticipated adaptation or scale recalibration? *Health Qual Life Outcomes*. 2013;11(1):1-10. doi:10.1186/1477-7525-11-171

Efanov JI, Nguyen DD, Izadpanah A, Danino MA, Harris P. A health utility assessment of trapeziectomy with ligament reconstruction and tendon interposition for thumb trapeziometacarpal osteoarthritis. *J Hand Surg Eur Vol*. 2019;44(7):722-727. doi:10.1177/1753193419843850

Eldabe S, Lloyd A, Verdian L, et al. Eliciting health state utilities from the general public for severe chronic pain. *Eur J Heal Econ*. 2010;11(3):323-330. doi:10.1007/s10198-009-0178-x

Ernstsson O, Burström K, Heintz E, Mølsted Alvesson H. Reporting and valuing one's own health: a think aloud study using EQ-5D-5L, EQ VAS and a time trade-off question among patients with a chronic condition. Health Qual Life Outcomes. 2020;18(1):1-12. doi:10.1186/s12955-020-01641-4

Essink-Bot ML, Stuifbergen MC, Meerding WJ, et al. Individual differences in the use of the response scale determine valuations of hypothetical health states: An empirical study. *BMC Health Serv Res*. 2007;7:1-10. doi:10.1186/1472-6963-7-62

Faris C, Heiser A, Quatela O, et al. Health utility of rhinectomy, surgical nasal reconstruction, and prosthetic rehabilitation. *Laryngoscope*. 2020;130(7):1674-1679. doi:10.1002/lary.28480

Faris C, Tessler O, Heiser A, Hadlock T, Jowett N. Evaluation of societal health utility of facial palsy and facial reanimation. *JAMA Facial Plast Surg*. 2018;20(6):480-487. doi:10.1001/jamafacial.2018.0866

Fennessy FM, Kong CY, Tempany CM, Swan JS. Quality-of-life assessment of fibroid treatment options and outcomes. *Radiology*. 2011;259(3):785-792. doi:10.1148/radiol.11100704

Ference EH, Stubbs V, Lidder AK, et al. Measurement and comparison of health utility assessments in chronic rhinosinusitis. *Int Forum Allergy Rhinol*. 2015;5(10):929-936. doi:10.1002/alr.21556

Finger R, Hoffman AE, Fenwick EK. Patients' preferences in treatment for neovascular age-related macular degeneration in clinical routine. *Br J Ophthalmol*. 2012;96(9):1276. doi:10.1136/bjophthalmol-2011-301201corr1

Fisman DN. Health related quality of life in genital herpes: A pilot comparison of measures. *Sex Transm Infect*. 2005;81(3):267-270. doi:10.1136/sti.2004.011619

Flood EM, De Cock E, Mörk AC, Revicki DA. Evaluating preference weights for the Asthma Symptom Utility Index (ASUI) across countries. *Health Qual Life Outcomes*. 2006;4:1-10. doi:10.1186/1477-7525-4-51

Foster C, Baki J, Nikirk S, et al. Comprehensive Health-State Utilities in Contemporary Patients With Cirrhosis. *Hepatol Commun*. 2020;4(6):852-858. doi:10.1002/hep4.1512

Frederix GWJ, Quadri N, Hövels AM, et al. Utility and Work Productivity Data for Economic Evaluation of Breast Cancer Therapies in the Netherlands and Sweden. *Clin Ther*. 2013;35(4):e1-e7. doi:10.1016/j.clinthera.2013.03.009

Gandhi M, Thumboo J, Luo N, Wee HL, Cheung YB. Do chronic disease patients value generic health states differently from individuals with no chronic disease? A case of a multicultural Asian population. *Health Qual Life Outcomes*. 2015;13(1). doi:10.1186/s12955-014-0200-6

Gandhi M, Thumboo J, Wee HL, Luo N, Cheung YB. How is the most severe health state being valued by the general population? *Health Qual Life Outcomes*. 2014;12(1):1-7. doi:10.1186/s12955-014-0161-9

Gaskin DJ, Frick KD. Race and ethnic disparities in valuing health. *Med Decis Mak*. 2008;28(1):12-20. doi:10.1177/0272989X07309641

Glick HA, Polsky D, Willke RJ, Schulman KA. A comparison of preference assessment instruments used in a clinical trial: Responses to the visual analog scale from the EuroQol EQ-5D and the Health Utilities Index. *Med Decis Mak*. 1999;19(3):265-275. doi:10.1177/0272989X9901900305

Golicki D, Jakubczyk M, Niewada M, Wrona W, Busschbach JJV. Valuation of EQ-5D health states in Poland: First TTO-based social value set in central and Eastern Europe. *Value Heal*. 2010;13(2):289-297. doi:10.1111/j.1524-4733.2009.00596.x

Goudarzi R, Zeraati H, Sari AA, Rashidian A, Mohammad K. Population-based preference weights for the EQ-5D health states using the visual analogue scale (VAS) in Iran. *Iran Red Crescent Med J*. 2016;18(2). doi:10.5812/ircmj.21584

Green C, Kiebert G, Murphy C, et al. Patients' health-related quality-of-life and health state values for motor neurone disease/amyotrophic lateral sclerosis. *Qual Life Res*. 2003;12(5):565-574. doi:10.1023/A:1025052609818

Greenleaf EK, Winder JS, Hollenbeak CS, et al. Cost-effectiveness of per oral endoscopic myotomy relative to laparoscopic Heller myotomy for the treatment of achalasia. *Surg Endosc*. 2018;32(1):39-45. doi:10.1007/s00464-017-5629-3

Greiner W, Claes C, Busschbach JJV, Graf Von Der Schulenburg JM. Validating the EQ-5D with time trade off for the German population. *Eur J Heal Econ*. 2005;6(2):124-130. doi:10.1007/s10198-004-0264-z

Greiner W, Weijnen T, Nieuwenhuizen M, et al. A single European currency for EQ-5D health states: Results from a six-country study. *Eur J Heal Econ*. 2003;4(3):222-231. doi:10.1007/s10198-003-0182-5

Grunberg SM, Boutin N, Ireland A, Miner S, Silveira J, Ashikaga T. Impact of nausea/vomiting on quality of life as a visual analogue scale-derived utility score. *Support Care Cancer*. 1996;4(6):435-439. doi:10.1007/BF01880641

Gudex C, Dolan P, Kind P, Williams A. Health state valuations from the general public using the visual analogue scale. *Qual Life Res*. 1996;5(6):521-531. doi:10.1007/BF00439226

Günther OH, Roick C, Angermeyer MC, König HH. The responsiveness of EQ-5D utility scores in patients with depression: A comparison with instruments measuring quality of life, psychopathology and social functioning. *J Affect Disord*. 2008;105(1-3):81-91. doi:10.1016/j.jad.2007.04.018

Gutacker N, Patton T, Shah K, Parkin D. Using EQ-5D Data to Measure Hospital Performance: Are General Population Values Distorting Patients' Choices? *Med Decis Mak*. 2020;40(4):511-521. doi:10.1177/0272989X20927705

Hakim Z, Wolf A, Garrison LP. Estimating the effect of changes in body mass index on health state preferences. *Pharmacoeconomics*. 2002;20(6):393-404. doi:10.2165/00019053-200220060-00004

Hanmer J, Cella D, Feeny D, et al. Evaluation of options for presenting health-states from PROMIS® item banks for valuation exercises. *Qual Life Res*. 2018;27(7):1835-1843. doi:10.1007/s11136-018-1852-1

Hanmer J, Cherepanov D, Palta M, et al. Health condition impacts in a nationally representative cross-sectional survey vary substantially by preference-based health index. *Med Decis Mak*. 2016;36(2):264-274. doi:10.1177/0272989X15599546

Happich M, Von Lengerke T. Valuing the health state "tinnitus": Differences between patients and the general public. *Hear Res*. 2005;207(1-2):50-58. doi:10.1016/j.heares.2005.04.002

Harijee A, Thankappan K, Mathew J, Sharma M, Iyer S. Comparison of health state utility measures and disabilities of arm, shoulder and hand questionnaire scores in bilateral hand amputees. J Plast Reconstr Aesthetic Surg. 2022;75(3):980-990. doi:10.1016/j.bjps.2021.11.061

Harvie HS, Arya LA, Saks EK, et al. Utility preference score measurement in women with fecal incontinence. *Am J Obstet Gynecol*. 2011;204(1):72.e1-72.e6. doi:10.1016/j.ajog.2010.08.018

Harvie HS, Lee DD, Andy UU, Shea JA, Arya LA. Validity of utility measures for women with pelvic organ prolapse. *Am J Obstet Gynecol*. 2018;218(1):119.e1-119.e8. doi:10.1016/j.ajog.2017.09.022

Harvie HS, Shea JA, Andy UU, et al. Validity of utility measures for women with urge, stress, and

mixed urinary incontinence. *Am J Obstet Gynecol*. 2014;210(1):85.e1-85.e6. doi:10.1016/j.ajog.2013.09.025

Havranek EP, Simon TA, L'Italien G, et al. The relationship between health perception and utility in heart failure patients in a clinical trial: Results from an OVERTURE substudy. *J Card Fail*. 2004;10(4):339-343. doi:10.1016/j.cardfail.2003.11.002

Havrilesky LJ, Broadwater G, Davis DM, et al. Determination of quality of life-related utilities for health states relevant to ovarian cancer diagnosis and treatment. *Gynecol Oncol*. 2009;113(2):216-220. doi:10.1016/j.ygyno.2008.12.026

Heintz E, Wiréhn AB, Peebo BB, Rosenqvist U, Levin LÅ. QALY weights for diabetic retinopathy - A comparison of health state valuations with HUI-3, EQ-5D, EQ-VAS, and TTO. *Value Heal*. 2012;15(3):475-484. doi:10.1016/j.jval.2011.11.031

Heiser A, Jowett N, Occhiogrosso J, Tessler O, Tan OT. Societal-perceived health utility of hypertrophic facial port-wine stain and laser treatment. *Facial Plast Surg Aesthetic Med*. 2020;22(5):327-335. doi:10.1089/fpsam.2020.0059

Hong J. Relative importance of the EQ-5D five dimensions among patients with chronic diseases in South Korea - a comparison with the general population preference weights. *Health Qual Life Outcomes*. 2018;16(1):1-10. doi:10.1186/s12955-018-0987-7

Hoots WK, Ebbesen LS, Konkle BA, et al. Secondary prophylaxis with recombinant activated factor VII improves health-related quality of life of haemophilia patients with inhibitors. *Haemophilia*. 2008;14(3):466-475. doi:10.1111/j.1365-2516.2008.01654.x

Horowitz E, Abadi-Korek I, Shani M, Shemer J. EQ-5D as a generic measure of health-related quality of life in Israel: reliability, validity and responsiveness. *Isr Med Assoc J*. 2010;12(12):715-720.

Huber FL, Furian M, Kohler M, et al. Health Preference Measures in Patients with Obstructive Sleep Apnea Syndrome Undergoing Continuous Positive Airway Pressure Therapy: Data from a Randomized Trial. Respiration. 2021;100(4):328-338. doi:10.1159/000513306

Huber M, Vogelmann M, Leidl R. Valuing health-related quality of life: Systematic variation in health perception. *Health Qual Life Outcomes*. 2018;16(1):1-9. doi:10.1186/s12955-018-0986-8

Ibrahim AMS, Sinno HH, Izadpanah A, et al. Mastopexy for breast ptosis: Utility outcomes of population preferences. *Can J Plast Surg*. 2015;23(2):103-107. doi:10.1177/229255031502300210

Ibrahim AMS, Sinno HH, Izadpanah A, et al. Population preferences of undergoing brachioplasty for arm laxity. *Ann Plast Surg*. 2014;73(December):S149-S152. doi:10.1097/SAP.0000000000000131

Insinga RP, Fryback DG. Understanding differences between self-ratings and population ratings for health in the EuroQOL. *Qual Life Res*. 2003;12(6):611-619. doi:10.1023/A:1025170308141

Iqbal I, Dasgupta B, Taylor P, Heron L, Pilling C. Elicitation of health state utilities associated with differing durations of morning stiffness in rheumatoid arthritis. *J Med Econ*. 2012;15(6):1192-1200. doi:10.3111/13696998.2012.712927

Izadpanah A, Sinno H, Vorstenbosch J, Lee BT, Lin SJ. Thigh laxity after massive weight loss: A

utilities outcomes assessment. *Ann Plast Surg*. 2013;71(3):304-307. doi:10.1097/SAP.0b013e3182834b88

Janssen MF, Birnie E, Bonsel GJ. Quantification of the level descriptors for the standard EQ-5D three-level system and a five-level version according to two methods. *Qual Life Res*. 2008;17(3):463-473. doi:10.1007/s11136-008-9318-5

Jansen SJT, Stiggelbout AM, Nooij MA, Kievit J. The effect of individually assessed preference weights on the relationship between holistic utilities and nonpreference-based assessment. Qual Life Res. 2000;9(5):541-557. doi:10.1023/A:1008917432368

Jansen SJT, Kievit J, Nooij MA, Stiggelbout AM. Stability of patients' preferences for chemotherapy: The impact of experience. Med Decis Mak. 2001;21(4):295-306. doi:10.1177/02729890122062596

Jia TW, Utzinger J, Deng Y, et al. Quantifying quality of life and disability of patients with advanced schistosomiasis japonica. *PLoS Negl Trop Dis*. 2011;5(2). doi:10.1371/journal.pntd.0000966

Jia TW, Zhou XN, Wang XH, et al. Assessment of the age-specific disability weight of chronic schistosomiasis japonica. *Bull World Health Organ*. 2007;85(6):458-465. doi:10.2471/BLT.06.033035

Jo MW, Lee HJ, Ryu DR, et al. Estimation of population-based utility weights for health states of chronic kidney disease, dialysis and kidney transplantation. *Nephrology*. 2020;25(7):544-550. doi:10.1111/nep.13700

Jo MW, Yun SC, Lee S Il. Estimating quality weights for EQ-5D health states with the time trade-off method in South Korea. *Value Heal*. 2008;11(7):1186-1189. doi:10.1111/j.1524-4733.2008.00348.x

Johnson AR, Asban A, Granoff MD, et al. Is Immediate Lymphatic Reconstruction Cost-effective? *Ann Surg*. 2019;274(6):E581-E588. doi:10.1097/SLA.0000000000003746

Johnson JA, Coons SJ, Ergo A, Szava-Kovats G. Valuation of EuroQOL (EQ-5D) health states in an adult US sample. *Pharmacoeconomics*. 1998;13(4):421-433. doi:10.2165/00019053-199813040-00005

Johnson JA, Ohinmaa A, Murti B, Sintonen H, Coons SJ. Comparison of Finnish and U.S.-based visual analog scale valuations of the EQ-5D measure. *Med Decis Mak*. 2000;20(3):281-289. doi:10.1177/0272989X0002000304

Johnston K, Brown J, Gerard K, O'Hanlon M, Morton A. Valuing temporary and chronic health states associated with breast screening. *Soc Sci Med*. 1998;47(2):213-222. doi:10.1016/S0277-9536(98)00065-3

Joyce VR, Barnett PG, Bayoumi AM, et al. Health-related quality of life in a randomized trial of antiretroviral therapy for advanced HIV disease. *J Acquir Immune Defic Syndr*. 2009;50(1):27-36. doi:10.1097/QAI.0b013e31818ce6f3

Joyce VR, Barnett PG, Chow A, et al. Effect of treatment interruption and intensification of antiretroviral therapy on health-related quality of life in patients with advanced HIV: A randomized, controlled trial. *Med Decis Mak*. 2012;32(1):70-82. doi:10.1177/0272989X10397615

Kang HJ, Kang E, Jo MW, et al. The utility score of epilepsy with partial seizure measured by TTO, VAS, and EQ-5D in the general Korean population. *Epilepsy Res*. 2014;108(5):963-971. doi:10.1016/j.eplepsyres.2014.02.014

Kangwanrattanakul K, Gross CR, Sunantiwat M, Thavorncharoensap M. Adding two culture-specific

'bolt-on' dimensions on the Thai version of EQ-5D-5L: an exploratory study in patients with diabetes. Expert Rev *Pharmacoeconomics Outcomes Res*. 2019;19(3):321-329. doi:10.1080/14737167.2019.1525294

Kangwanrattanakul K, Gross CR, Sunantiwat M, Thavorncharoensap M. Exploration of a cultural-adaptation of the EQ-5D for Thai population: A "bolt-on" experiment. *Qual Life Res*. 2019;28(5):1207-1215. doi:10.1007/s11136-018-2072-4

Kartman B, Gatz G, Johannesson M. Health State Utilities in Gastroesophageal Reflux Disease Patients with Heartburn: A Study in Germany and Sweden. *Med Decis Mak*. 2004;24(1):40-52. doi:10.1177/027298X03261563

Kerrigan CL, Dale Collins E, Kneeland TS, et al. Measuring health state preferences in women with breast hypertrophy. *Plast Reconstr Surg*. 2000;106(2):280-288. doi:10.1097/00006534-200008000-00005

Khabibullina A, Gerry CJ. Valuing Health States in Russia: A First Feasibility Study. *Value Heal Reg Issues*. 2019;19:75-80. doi:10.1016/j.vhri.2019.01.005

Khanna D, Ahmed M, Furst DE, et al. Health values of patients with systemic sclerosis. *Arthritis Care Res*. 2007;57(1):86-93. doi:10.1002/art.22465

Kiebert GM, Green C, Murphy C, et al. Patients' health-related quality of life and utilities associated with different stages of amyotrophic lateral sclerosis. *J Neurol Sci*. 2001;191(1-2):87-93. doi:10.1016/S0022-510X(01)00616-5

Kim EJ, Ock M, Kim KP, et al. Disease severity-based evaluation of utility weights for lung cancer-related health states in Korea. *BMC Cancer*. 2018;18(1):1-8. doi:10.1186/s12885-018-4960-y

Kim S, Jo M, Lee H, Lee J. Estimation of health state utilities in breast cancer. *Patient Pref Adh* 2017:531-536.

Kim SH, Lee S il, Jo MW. Feasibility, comparability, and reliability of the standard gamble compared with the rating scale and time trade-off techniques in Korean population. *Qual Life Res*. 2017;26(12):3387-3397. doi:10.1007/s11136-017-1676-4

Kind P, Klose K, Gusi N, Olivares PR, Greiner W. Can adult weights be used to value child health states? Testing the influence of perspective in valuing EQ-5D-Y. *Qual Life Res*. 2015;24(10):2519-2539. doi:10.1007/s11136-015-0971-1

King JT, Styn MA, Tsevat J, Roberts MS. "Perfect health" versus "disease free": The impact of anchor point choice on the measurement of preferences and the calculation of disease-specific disutilities. *Med Decis Mak*. 2003;23(3):212-225. doi:10.1177/0272989X03023003003

King JT, Tsevat J, Roberts MS. Cognitive impairment and preferences for current health. *Health Qual Life Outcomes*. 2009;7:1-9. doi:10.1186/1477-7525-7-1

King JT, Tsevat J, Roberts MS. Positive association between current health and health values for hypothetical disease states. *Med Decis Mak*. 2004;24(4):367-378. doi:10.1177/0272989X04267692

Kirigia JM. Economic evaluation in schistosomiasis: Valuation of health states preferences. *Health Econ*. 1998;7(6):551-556. doi:10.1002/(SICI)1099-1050(199809)7:6<551::AID-HEC367>3.0.CO;2-7

Knies S, Boonen A, Severens JL. Do the Washington Panel recommendations hold for

Europe: Investigating the relation between quality of life versus work-status, absenteeism and presenteeism. *Cost Eff Resour Alloc*. 2014;12(1):1-8. doi:10.1186/1478-7547-12-24

Ko WC, Tsai TF, Tang CH. Health state utility, willingness to pay, and quality of life among Taiwanese patients with psoriasis. *Dermatologica Sin*. 2016;34(4):185-191. doi:10.1016/j.dsi.2016.05.004

Kohlmann T, Wang C, Lipinski J, et al. The impact of a patient support program for multiple sclerosis on patient satisfaction and subjective health status. *J Neurosci Nurs*. 2013;45(3). doi:10.1097/JNN.0b013e31828a4161

Korfage IJ, De Koning HJ, Habbema JDF, Schröder FH, Essink-Bot ML. Side-effects of treatment for localized prostate cancer: Are they valued differently by patients and healthy controls? *BJU Int*. 2007;99(4):801-806. doi:10.1111/j.1464-410X.2006.06707.x

Krabbe PFM, Salomon JA, Murray CJL. Quantification of health states with rank-based nonmetric multidimensional scaling. *Med Decis Mak*. 2007;27(4):395-405. doi:10.1177/0272989X07302131

Krabbe PFM, Stalmeier PFM, Lamers LM, Busschbach JJV. Testing the interval-level measurement property of multi-item visual analogue scales. *Qual Life Res*. 2006;15(10):1651-1661. doi:10.1007/s11136-006-0027-7

Krabbe PFM, Stolk EA, Devlin NJ, et al. Head-to-head comparison of health-state values derived by a probabilistic choice model and scores on a visual analogue scale. *Eur J Heal Econ*. 2017;18(8):967-977. doi:10.1007/s10198-016-0841-y

Krabbe PFM. Thurstone scaling as a measurement method to quantify subjective health outcomes. *Med Care*. 2008;46(4):357-365. doi:10.1097/MLR.0b013e31815ceca9

Kraemer KL, Roberts MS, Horton NJ, et al. Health utility ratings for a spectrum of alcohol-related health states. *Med Care*. 2005;43(6):541-550. doi:10.1097/01.mlr.0000163644.97251.14

Krol M, Brouwer W, Sendi P. Productivity Costs in Health-State Valuations. *Pharmacoeconomics*. 2006;24(4):401-414. doi:10.2165/00019053-200624040-00009

Krol M, Nap A, Michels R, Veraart C, Goossens L. Health state utilities for infertility and subfertility. *Reprod Health*. 2019;16(1):1-9. doi:10.1186/s12978-019-0706-9

Kuta V, McNeely PD, Walling S, Bezuhly M. Sagittal craniosynostosis: A utility outcomes study. *J Neurosurg Pediatr*. 2017;20(2):113-118. doi:10.3171/2017.2.PEDS16567

Lamers LM, Stalmeier PFM, Krabbe PFM, Busschbach JJV. Inconsistencies in TTO and VAS values for EQ-5D health states. *Med Decis Mak*. 2006;26(2):173-181. doi:10.1177/0272989X06286480

Langfitt JT, Vickrey BG, McDermott MP, et al. Validity and responsiveness of generic preference-based HRQOL instruments in chronic epilepsy. *Qual Life Res*. 2006;15(5):899-914. doi:10.1007/s11136-005-5231-3

Lara AM, Wakholi BN, Kasirye A, et al. Utility assessment of HIV/AIDS-related health states in HIV-infected Ugandans. *AIDS*. 2008;22 Suppl 1:123-130. doi:10.1097/01.aids.0000327633.85221.9a

Lavelle TA, Crossnohere NL, Bridges JFP. Quantifying the Burden of Hyperphagia in Prader-Willi Syndrome Using Quality-Adjusted Life-years.

Clin Ther. 2021;43(7):1164-1178.e4. doi:10.1016/j.clinthera.2021.05.013

Lee HY, Park EC, Joong Kim H, Choi JY, Kim HN. Cost-utility analysis of cochlear implants in Korea using different measures of utility. *Acta Otolaryngol*. 2006;126(8):817-823. doi:10.1080/00016480500525213

Lee TA, Hollingworth W, Sullivan SD. Comparison of directly elicited preferences to preferences derived from the SF-36 in adults with asthma. *Med Decis Mak*. 2003;23(4):323-334. doi:10.1177/0272989X03256009

Leidl R, Reitmeir P. A value set for the EQ-5D based on experienced health states: Development and testing for the German population. *Pharmacoeconomics*. 2011;29(6):521-534. doi:10.2165/11538380-000000000-00000

Leidl R, Reitmeir P. An Experience-Based Value Set for the EQ-5D-5L in Germany. *Value Heal*. 2017;20(8):1150-1156. doi:10.1016/j.jval.2017.04.019

Leidl R, Schweikert B, Hahmann H, Steinacker JM, Reitmeir P. Assessing quality of life in a clinical study on heart rehabilitation patients: How well do value sets based on given or experienced health states reflect patients' valuations? *Health Qual Life Outcomes*. 2016;14(1):1. doi:10.1186/s12955-016-0453-3

Lenert LA, Morss S, Goldstein MK, et al. Measurement of the Validity of Utility Elicitations Performed by Computerized Interview. *Med Care*. 1997;35(9):915-920. doi:10.1097/00005650-199709000-00004

Lenert LA, Sturley AE. Acceptability of computerized visual analog scale, time trade-off and standard gamble rating methods in patients and the public. *Proc AMIA Symp*. 2001:364-368.

Lenert LA, Sturley AP, Rapaport MH, et al. Public preferences for health states with schizophrenia and a mapping function to estimate utilities from positive and negative symptom scale scores. *Schizophr Res*. 2004;71(1):155-165. doi:10.1016/j.schres.2003.10.010

Lenert LA, Treadwell JR. Effects on preferences of violations of procedural invariance. *Med Decis Mak*. 1999;19(4):473-481. doi:10.1177/0272989X9901900415

Lenert LA, Ziegler J, Lee T, Sommi R, Mahmoud R. Differences in health values among patients, family members, and providers for outcomes in schizophrenia. *Med Care*. 2000;38(10):1011-1021. doi:10.1097/00005650-200010000-00005

Lenert LA, Ziegler J, Lee T, Unfred C, Mahmoud R. The Risks of Multimedia Methods : *J Am Med Informatics Assoc*. 2000;7(2):177-185.

Leoncini E, Ricciardi W, Cadoni G, et al. Adult height and head and neck cancer: A pooled analysis within the INHANCE Consortium. *Head Neck*. 2014;36(10):1391. doi:10.1002/HED

Levo H, Stephens D, Poe D, et al I. EuroQol 5D quality of life in Menière's disorder can be explained with symptoms and disabilities. *Int J Rehabil Res*. 2012;35(3):197-202. doi:10.1097/MRR.0b013e3283531f95

Lipman SA, Reckers-Droog VT, Karimi M, Jakubczyk M, Attema AE. Self vs. other, child vs. adult. An experimental comparison of valuation perspectives for valuation of EQ-5D-Y-3L health states. Eur J Heal Econ. 2021;22(9):1507-1518. doi:10.1007/s10198-021-01377-y

Lloyd A, Van Hanswijck De Jonge P, Doyle S, Cornes P. Health state utility scores for cancer-related anemia through societal and patient

valuations. *Value Heal*. 2008;11(7):1178-1185. doi:10.1111/j.1524-4733.2008.00394.x

Lo SH, Lloyd A, Marshall J, Vyas K. Patient and Caregiver Health State Utilities in Lennox-Gastaut Syndrome and Dravet Syndrome. Clin Ther. 2021;43(11):1861-1876.e16. doi:10.1016/j.clinthera.2021.09.017

Loon YV, Stiggelbout AM, Hakkesteegt MM, et al. Utility approach to decision-making in extended T1 and limited T2 glottic carcinoma. *J Sci Spec Head Neck*. 2017;36(10):1391. doi:10.1002/HED

Lundberg L, Johannesson M, Silverdahl M, Hermansson C, Lindberg M. Quality of life, health-state utilities and willingness to pay in patients with psoriasis and atopic eczema. *Br J Dermatol*. 1999;141(6):1067-1075. doi:10.1046/j.1365-2133.1999.03207.x

Lundsberg LS, Xu X, Schwarz EB, Gariepy AM. Measuring health utility in varying pregnancy contexts among a diverse cohort of pregnant women. *Contraception*. 2017;96(6):411-419. doi:10.1016/j.contraception.2017.08.003

Lyerly AD, Myers ER, Faden RR. The ethics of aggregation and hormone replacement therapy. *Heal Care Anal*. 2001;9(2):187-211. doi:10.1023/A:1011386320609

Macran S, Kind P. "Death" and the Valuation of Health-Related Quality of Life. *Med Care*. 2001;39(3):217-227. doi:10.1097/00005650-200103000-00003

Makhdom AM, Sinno H, Aldebeyan S, et al. Bilateral Hallux Valgus: A Utility Outcome Score Assessment. *J Foot Ankle Surg*. 2016;55(5):944-947. doi:10.1053/j.jfas.2016.04.010

Mann, Rachel; Brazier, John; Tsuchiya A. A Comparison of patient and general population weightings of EQ-5D dimensions. *Health Econ*. 2008;1131(2007):1127-1131. doi:10.1002/hec

McCabe C, Stevens K, Roberts J, Brazier J. Health state values for the HUI 2 descriptive system: Results from a UK survey. *Health Econ*. 2005;14(3):231-244. doi:10.1002/hec.925

McCarthy AE, Coyle D. Determining utility values related to malaria and malaria chemoprophylaxis. *Malar J*. 2010;9(1):1-5. doi:10.1186/1475-2875-9-92

McDonough CM, Grove MR, Tosteson TD, et al. Comparison of EQ-5D, HUI, and SF-36-derived societal health state values among Spine Patient Outcomes Research Trial (SPORT) participants. *Qual Life Res*. 2005;14(5):1321-1332. doi:10.1007/s11136-004-5743-2

McPhail S, Beller E, Haines T. Reference bias: Presentation of extreme health states prior to eq-vas improves health-related quality of life scores. A randomised cross-over trial. *Health Qual Life Outcomes*. 2010;8:1-11. doi:10.1186/1477-7525-8-146

McPherson K, Myers J, Taylor WJ, McNaughton HK, Weatherall M. Self-valuation and societal valuations of health state differ with disease severity in chronic and disabling conditions. *Med Care*. 2004;42(11):1143-1151. doi:10.1097/00005650-200411000-00014

Md Yusof FA, Goh A, Azmi S. Estimating an EQ-5D value set for malaysia using time trade-off and visual analogue scale methods. *Value Heal*. 2012;15(1 SUPPL.):S85-S90. doi:10.1016/j.jval.2011.11.024

Michel YA, Augestad LA, Barra M, Rand K. A Norwegian 15D value algorithm: proposing a new procedure to estimate 15D value algorithms. Qual

Life Res. 2019;28(5):1129-1143. doi:10.1007/s11136-018-2043-9

Milne RJ, Heaton-Brown KH, Hansen P, et al. Quality-of-life valuations of advanced breast cancer by New Zealand women. *Pharmacoeconomics*. 2006;24(3):281-292. doi:10.2165/00019053-200624030-00007

Mohide EA, Archibald SD, Tew M, Young JE, Haines T. Postlaryngectomy quality-of-life dimensions identified by patients and health care professionals. *Am J Surg*. 1992;164(6):619-622. doi:10.1016/S0002-9610(05)80720-2

Moore AD, Clarke AE, Danoff DS, Joseph L, Bélisle P, Neville C, Fortin PR. Can health utility measures be used in lupus research? A comparative validation and reliability study of 4 utility indices. *J Rheumatol*. 1999;26(6):1285-90.

Montejo ÁL, Correas-Lauffer J, Mauriño J, et al. Estimation of a multiattribute utility function for the Spanish version of the tool questionnaire. *Value Heal*. 2011;14(4):564-570. doi:10.1016/j.jval.2010.11.016

Moradi N, Woldemichael A, Malekian P, Rotvandi DM, Rezaei S. An exploratory study to estimate cost-effectiveness threshold value for life saving treatments in western Iran. Cost Eff Resour Alloc. 2020;18(1):1-9. doi:10.1186/s12962-020-00241-9

Nemes S, Burström K, Zethraeus N, et al. Assessment of the Swedish EQ-5D experience-based value sets in a total hip replacement population. *Qual Life Res*. 2015;24(12):2963-2970. doi:10.1007/s11136-015-1020-9

Nimdet K, Ngorsuraches S. Willingness to pay per quality-adjusted life year for life-saving treatments in Thailand. *BMJ Open*. 2015;5(10):1-7. doi:10.1136/bmjopen-2015-008123

Noel CW, Lee DJ, Kong Q, et al. Comparison of health state utility measures in patients with head and neck cancer. *JAMA Otolaryngol - Head Neck Surg*. 2015;141(8):696-703. doi:10.1001/jamaoto.2015.1314

Nord E. EuroQol©: health-related quality of life measurement. Valuations of health states by the general public in Norway. *Health Policy*. 1991;18(1):25-36. doi:10.1016/0168-8510(91)90141-J

Nord E. The validity of a visual analogue scale in determining social utility weights for health states. *Int J Health Plann Manage*. 1991;6(3):234-242. doi:10.1002/hpm.4740060308

Nuyen B, Kandathil C, McDonald D, Chou DW, Shih C, Most SP. The Health Burden of Transfeminine Facial Gender Dysphoria: An Analysis of Public Perception. Facial Plast Surg Aesthetic Med. 2021;23(5):350-356. doi:10.1089/fpsam.2020.0192

Nuyen B, Kandathil C, McDonald D, Thomas J, Most SP. The impact of living with transfeminine vocal gender dysphoria: Health utility outcomes assessment. Int J Transgender Heal. 2021;0(0):1-9. doi:10.1080/26895269.2021.1919277

Ock M, Ahn J, Yoon SJ, Jo MW. Estimation of disability weights in the general population of South Korea using a paired comparison. *PLoS One*. 2016;11(9):1-25. doi:10.1371/journal.pone.0162478

Ock M, Lim SY, Lee HJ, Kim SH, Jo MW. Estimation of utility weights for major liver diseases according to disease severity in Korea. *BMC Gastroenterol*. 2017;17(1):1-7. doi:10.1186/s12876-017-0660-3

Ock M, Park JY, Son WS, Lee HJ, Kim SH, Jo MW. Estimation of utility weights for human

papilloma virus-related health states according to disease severity. *Health Qual Life Outcomes*. 2016;14(1). doi:10.1186/s12955-016-0566-8

Oddershede L, Andreasen JJ, Ehlers L. Estimation of utility values from visual analog scale measures of health in patients undergoing cardiac surgery. *Clin Outcomes Res*. 2014;6(1):21-27. doi:10.2147/CEOR.S55899

Ohsawa I, Ishida T, Oshida Y, Yamanouchi K, Sato Y. Subjective health values of individuals with diabetes in Japan: Comparison of utility values with the SF-36 scores. *Diabetes Res Clin Pract*. 2003;62(1):9-16. doi:10.1016/S0168-8227(03)00145-1

Olsen JA, Lindberg MH, Lamu AN. Health and wellbeing in Norway: Population norms and the social gradient. Soc Sci Med. 2020;259(July):113155. doi:10.1016/j.socscimed.2020.113155

Patterson D, Geisler BP, Morse A. Determining health-related quality of life and health state utility values of urinary incontinence in women. *J Female Pelvic Med Reconstr Surg*. 2011;26(5):287-298. doi:10.1097/SPV

Perneger T V., Courvoisier DS. Exploration of health dimensions to be included in multi-attribute health-utility assessment. *Int J Qual Heal Care*. 2011;23(1):52-59. doi:10.1093/intqhc/mzq068

Pickard AS, Hung YT, Lin FJ, Lee TA. Patient Experience-based Value Sets: Are They Stable? *Medical Care*. 2017;55(11), 979–984. Doi:10.1097/MLR.0000000000000802

Pisa G, Reinhold T, Obi-Tabot E, Bodoria M, Brüggenjürgen B. Critical limb ischemia and its impact on patient health preferences and quality of lifean international study. *Int J Angiol*. 2012;21(3):139-146. doi:10.1055/s-0032-1324738

Poenaru D, Pemberton J, Frankfurter C, Cameron BH, Stolk E. Establishing disability weights for congenital pediatric surgical conditions: A multi-modal approach. *Popul Health Metr*. 2017;15(1):1-11. doi:10.1186/s12963-017-0125-5

Polsky D, Keating NL, Weeks JC, Schulman KA. Patient choice of breast cancer treatment: Impact on health state preferences. *Med Care*. 2002;40(11):1068-1079. doi:10.1097/00005650-200211000-00008

Prevolnik Rupel V, Srakar A, Rand K. Valuation of EQ-5D-3l health states in Slovenia: VAS based and TTO based Value Sets. *Zdr Varst*. 2020;59(1):8-17. doi:10.2478/sjph-2020-0002

Raat H, Bonsel GJ, Hoogeveen WC, et al. Feasibility and reliability of a mailed questionnaire to obtain visual analogue scale valuations for health states defined by the health utilities index mark 3. *Med Care*. 2004;42(1):13-18. doi:10.1097/01.mlr.0000102297.06535.e7

Ramakers GGJ, Smulders YE, van Zon A, et al. Agreement between health utility instruments in cochlear implantation. *Clin Otolaryngol*. 2016;41(6):737-743. doi:10.1111/coa.12626

Rashidi AA, Anis AH, Marra CA. Do visual analogue scale (VAS) derived standard gamble (SG) utilities agree with Health Utilities Index utilities? A comparison of patient and community preferences for health status in rheumatoid arthritis patients. *Health Qual Life Outcomes*. 2006;4:1-10. doi:10.1186/1477-7525-4-25

Rathore SS, Lenert LA, Weinfurt KP, et al. The effects of patient sex and race on medical students' ratings of quality of life. *Am J Med*. 2000;108(7):561-566. doi:10.1016/S0002-9343(00)00352-1

Raymakers AJN, Tsao NW, Marra CA, Clements PJ, Khanna D. Health state utilities and disease duration in systemic sclerosis: Is there an association? *J Rheumatol*. 2016;43(10):1832-1837. doi:10.3899/jrheum.160162

Reaney M, Stassek L, Martin M, et al. Creating a personalized evaluation framework for patient-reported outcomes: an illustration using the EQ-5D visual analogue scale. *Expert Rev Pharmacoeconomics Outcomes Res*. 2019;19(1):97-104. doi:10.1080/14737167.2019.1519398

Rencz F, Brodszky V, Stalmeier PFM, et al. Valuation of pemphigus vulgaris and pemphigus foliaceus health states: a convenience sample experiment. *Br J Dermatol*. 2016;175(3):593-599. doi:10.1111/bjd.14647

Retra JGA, Essers BAB, Joore MA, Evers SMAA, Dirksen CD. Age dependency of EQ-5D-Youth health states valuations on a visual analogue scale. Health Qual Life Outcomes. 2020;18(1):1-6. doi:10.1186/s12955-020-01638-z

Richardson J, Sinha K, Iezzi A, Khan MA. Modelling utility weights for the Assessment of Quality of Life (AQoL)-8D. *Qual Life Res*. 2014;23(8):2395-2404. doi:10.1007/s11136-014-0686-8

Robinson A, Loomes G, Jones-Lee M. Visual analog scales, standard gambles, and relative risk aversion. *Med Decis Mak*. 2001;21(1):17-27. doi:10.1177/0272989X0102100103

Rowen D, Brazier J, Tsuchiya A, Alava MH. Valuing states from multiplemeasures on the same visual analogue sale: a feasibility study. *Health Econ*. 2011;1131(2007):1127-1131. doi:10.1002/hec

Ruiz MA, Gutiérrez LL, Monroy M, Rejas J. Mapping of the OAB-SF Questionnaire onto EQ-5D in Spanish Patients with Overactive Bladder. *Clin Drug Investig*. 2016;36(4):267-279. doi:10.1007/s40261-016-0377-z

Rutten-Van Mölken MPMH, Hoogendoorn M, Lamers LM. Holistic preferences for 1-year health profiles describing fluctuations in health: The case of chronic obstructive pulmonary disease. *Pharmacoeconomics*. 2009;27(6):465-477. doi:10.2165/00019053-200927060-00003

Sanchez-Arenas R, Vargas-Alarcon G, Sanchez-Garcia S, et al. Value of EQ-5D in Mexican city older population with and without dementia (SADEM study). *Int J Geriatr Psychiatry*. 2014;29(5):478-488. doi:10.1002/gps.4030

Sano M, Albert SM, Tractenberg R, Schittini M. Developing Utilities: Quantifying Quality of Life for Stages of Alzheimer's Disease As Measured by the Clinical Dementia Rating. *J Ment Health Aging*. 1999;5:59-68.

Santos M, Monteiro AL, Santos B. Exploring the predictors of health valuations of EQ 5D 3L with a mixed-effects linear model. Expert Rev *Pharmacoeconomics Outcomes Res*. 2020;20(4):363-367. doi:10.1080/14737167.2019.1637734

Schmitt J, Meurer M, Klon M, Frick KD. Assessment of health state utilities of controlled and uncontrolled psoriasis and atopic eczema: A population-based study. *Br J Dermatol*. 2008;158(2):351-359. doi:10.1111/j.1365-2133.2007.08354.x

Schölzel-Dorenbos CJM, Arons AMM, Wammes JJG, Rikkert MGmo, Krabbe PFM. Validation study of the prototype of a disease-specific index measure for health-related quality of life in dementia. *Health Qual Life Outcomes*. 2012;10:1-11. doi:10.1186/1477-7525-10-118

Secnik K, Matza LS, Cottrell S, et al. Health state utilities for childhood attention-deficit/hyperactivity disorder based on parent preferences in the United Kingdom. *Med Decis Mak*. 2005;25(1):56-70. doi:10.1177/0272989X04273140

Seidl H, Hunger M, Leidl R, et al. Cost-effectiveness of nurse-based case management versus usual care for elderly patients with myocardial infarction: results from the KORINNA study. *Eur J Heal Econ*. 2015;16(6):671-681. doi:10.1007/s10198-014-0623-3

Sekigami Y, Char S, Mullen C, et al. Cost-Effectiveness Analysis: Lymph Node Transfer vs Lymphovenous Bypass for Breast Cancer-Related Lymphedema. J Am Coll Surg. 2021;232(6):837-845. doi:10.1016/j.jamcollsurg.2021.02.013

Sendi P, Brouwer WBF. Is silence golden? A test of the incorporation of the effects of ill-health on income and leisure in health state valuations. *Health Econ*. 2005;14(6):643-647. doi:10.1002/hec.971

Sengupta N, Nichol MB, Wu J, Globe D. Mapping the SF-12 to the HUI3 and VAS in a managed care population. *Med Care*. 2004;42(9):927-937. doi:10.1097/01.mlr.0000135812.52570.42

Shafie AA, Ng CH, Thanimalai S, Haron N, Manocha AB. Estimating the utility value of hypoglycaemia according to severity and frequency using the visual analogue scale (VAS) and time trade-off (TTO) survey. *J Diabetes Metab Disord*. 2018;17(2):269-275. doi:10.1007/s40200-018-0369-z

Shafie AA, Vasan Thakumar A. Multiplicative modelling of EQ-5D-3L TTO and VAS values. Eur J Heal Econ. 2020;21(9):1411-1420. doi:10.1007/s10198-020-01233-5

Shah KK, Ramos-Goñi JM, Kreimeier S, Devlin NJ. An exploration of methods for obtaining 0 = dead anchors for latent scale EQ-5D-Y values. *Eur J Heal Econ*. 2020;21(7):1091-1103. doi:10.1007/s10198-020-01205-9

Shahjouei S, Vafaei SA, Khorasani S, et al. Utility Measures in Pediatric Temporary Health States: Comparison of Prone Positioning Valuation Through 5 Assessment Tools. *Value Heal Reg Issues*. 2019;18:97-105. doi:10.1016/j.vhri.2019.01.003

Shaw RW, Brickley MR, Evans L, Edwards MJ. Perceptions of women on the impact of menorrhagia on their health using multi-attribute utility assessment. *BJOG An Int J Obstet Gynaecol*. 1998;105(11):1155-1159. doi:10.1111/j.1471-0528.1998.tb09968.x

Sheckter CC, Van Vliet MM, Krishnan NM, Garner WL. Cost-effectiveness comparison between topical silver sulfadiazine and enclosed silver dressing for partial-thickness burn treatment. *J Burn Care Res*. 2014;35(4):284-290. doi:10.1097/BCR.0b013e3182a36916

Shih V, Chan A, Xie F, Ko Y. Economic Evaluation of Anastrozole Versus Tamoxifen for Early Stage Breast Cancer in Singapore. *Value Heal Reg Issues*. 2012;1(1):46-53. doi:10.1016/j.vhri.2012.03.013

Shih V, Chan A, Xie F, Ko Y. Health State Utility Assessment for Breast Cancer. *Value Heal Reg Issues.* 2012;1(1):93-97. doi:10.1016/j.vhri.2012.03.009

Shmueli A, Messika D, Murad H, Freedman L. Does greater exposure to own-health data make a difference on the visual analog scale? *Eur J Heal Econ*. 2008;9(1):63-67. doi:10.1007/s10198-007-0040-y

Shmueli A. The visual analogy rating scale of health-related quality of life: An examination of end-digit preferences. *Health Qual Life Outcomes*. 2005;3:2-6. doi:10.1186/1477-7525-3-71

Simon PA, Hung YT, Lin FJ, Lee TA. Patient Experience-based Value Sets: Are They Stable? *Med Care*. 2017;55(11):979-984. doi:10.1097/MLR.0000000000000802

Sinno H, Izadpanah A, Tahiri Y, et al. The impact of living with severe lower extremity Lymphedema: A utility outcomes score assessment. *Ann Plast Surg*. 2014;73(2):210-214. doi:10.1097/SAP.0b013e3182713ce0

Sinno H, Izadpanah A, Thibaudeau S, et al. An objective assessment of the perceived quality of life of living with bilateral mastectomy defect. *Breast*. 2013;22(2):168-172. doi:10.1016/j.breast.2012.07.001

Sinno H, Izadpanah A, Thibaudeau S, et al. The impact of living with a functional and aesthetic nasal deformity after primary rhinoplasty: A utility outcomes score assessment. *Ann Plast Surg*. 2012;69(4):431-434. doi:10.1097/SAP.0b013e3182480384

Sinno H, Tahiri Y, Thibaudeau S, et al. Cleft lip and palate: An objective measure outcome study. *Plast Reconstr Surg*. 2012;130(2):408-414. doi:10.1097/PRS.0b013e3182589d4b

Sinno H, Thibaudeau S, Izadpanah A, et al. Utility outcome scores for unilateral facial paralysis. *Ann Plast Surg*. 2012;69(4):435-438. doi:10.1097/SAP.0b013e318246e698

Sinno H, Thibaudeau S, Tahiri Y, et al. Utility assessment of body contouring after massive weight loss. *Aesthetic Plast Surg*. 2011;35(5):724-730. doi:10.1007/s00266-011-9676-1

Sinno HH, Thibaudeau S, Duggal A, Lessard L. Utility scores for facial disfigurement requiring facial transplantation [Outcomes Article]. *Plast Reconstr Surg*. 2010;126(2):443-449. doi:10.1097/PRS.0b013e3181e094fa

Smith KJ, Roberts MS. Quality-of-life utility values for erectile function and sildenafil treatment. *Clin Drug Investig*. 2005;25(2):99-105. doi:10.2165/00044011-200525020-00002

Smith KJ, Tsevat J, Ness RB, Wiesenfeld HC, Roberts MS. Quality of life utilities for pelvic inflammatory disease health states. *Sex Transm Dis*. 2008;35(3):307-311. doi:10.1097/OLQ.0b013e31815b07dd

Song HJ, Park H, Park SY, et al. Estimation of Health Utilities Based on the Response to Treatment in Atopic Dermatitis: a Population-based Study. *Clin Ther*. 2019;41(4):700-713. doi:10.1016/j.clinthera.2019.02.007

Sonntag M, König HH, Konnopka A. The responsiveness of the EQ-5D and time trade-off scores in schizophrenia, affective disorders, and alcohol addiction. *Health Qual Life Outcomes*. 2015;13(1):1-9. doi:10.1186/s12955-015-0315-4

Stalmeier PFM, Buusschbach JJV, Lamers LM, Krabbe PFM. The gap effect: Discontinuities of preferences around dead. *Health Econ*. 2005;14(7):679-685. doi:10.1002/hec.986

Stalmeier PFM, De Boer AGEM, Sprangers MAG, De Haes HCJM, Van Lanschot JJB. Validity of utilities of patients with esophageal cancer. *Int J Technol Assess Health Care*. 2005;21(1):113-118. doi:10.1017/S0266462305050142

Stavem K. Quality of life in epilepsy: Comparison of four preference measures. *Epilepsy Res*. 1998;29(3):201-209. doi:10.1016/S0920-1211(97)00075-2

Stephens D, Pyykk I, Levo H, et al. Positive experiences and quality of life in Ménière's disorder. *Int J Audiol*. 2010;49(11):839-843. doi:10.3109/14992027.2010.510147

Stiggelbout AM, Eijkemans MJC, Kiebert GM, et al. The "utility" of the visual analog scale in medical decision making and technology assessment: Is it an alternative to the time trade-off? *Int J Technol Assess Health Care*. 1996;12(2):291-298. doi:10.1017/S0266462300009648

Suarez-Almazor ME, Conner-Spady B. Rating of arthritis health states by patients, physicians, and the general public. Implications for cost-utility analyses. *J Rheumatol*. 2001;28(3):648-656.

Sun S, Chen J, Kind P, et al. Experience-based VAS values for EQ-5D-3L health states in a national general population health survey in China. *Qual Life Res*. 2015;24(3):693-703. doi:10.1007/s11136-014-0793-6

Sun C, Brown AJ, Jhingran A, et al. Patient preferences for side effects associated with cervical cancer treatment. *Health Econ*. 2008;23(1):1-7. doi:10.1097/IGC.0000000000000149.Patient

Sung L, Greenberg ML, Young NL, et al. Validity of a modified standard gamble elicited from parents of a hospital-based cohort of children. *J Clin Epidemiol*. 2003;56(9):848-855. doi:10.1016/S0895-4356(03)00160-4

Svedbom A, Borgström F, Hernlund E, et al. Quality of life after hip, vertebral, and distal forearm fragility fractures measured using the EQ-5D-3L, EQ-VAS, and time-trade-off: results from the ICUROS. *Qual Life Res*. 2018;27(3):707-716. doi:10.1007/s11136-017-1748-5

Swan JS, Kong CY, Lee JM, et al. Patient and societal value functions for the testing morbidities index. *Med Decis Mak*. 2013;33(6):819-838. doi:10.1177/0272989X13487605

Szende A, Schaefer C, Goss TF, et al. Valuation of transfusion-free living in MDS: Results of health utility interviews with patients. *Health Qual Life Outcomes*. 2009;7:81. doi:10.1186/1477-7525-7-81

Takura T, Kawanishi H, Minakuchi J, Nagake Y, Takahashi S. Cost-effectiveness analysis of on-line hemodiafiltration in Japan. *Blood Purif*. 2013;35(SUPPL. 1):85-89. doi:10.1159/000346358

Tan XY, Aung MM, Ngai MI, Xie F, Ko Y. Assessment of preference for hormonal treatment-related health states among patients with breast cancer. *Value Heal Reg Issues*. 2014;3(1):27-32. doi:10.1016/j.vhri.2013.12.002

Teuffel O, Cheng S, Ethier MC, et al. Health-related quality of life anticipated with different management strategies for febrile neutropenia in adult cancer patients. *Support Care Cancer*. 2012;20(11):2755-2764. doi:10.1007/s00520-012-1397-8

Tongsiri S, Cairns J. Estimating population-based values for EQ-5D health states in Thailand. *Value Heal*. 2011;14(8):1142-1145. doi:10.1016/j.jval.2011.06.005

Torrance GW, Keresteci MA, Casey RW, et al. Development and initial validation of a new preference-based disease-specific health-related quality of life instrument for erectile function. *Qual Life Res*. 2004;13(2):349-359. doi:10.1023/B:QURE.0000018482.71580.f2

Trent M, Lehmann HP, Qian Q, et al. Adolescent and parental utilities for the health states associated with pelvic inflammatory disease. *Sex Transm Infect*. 2011;87(7):583-587. doi:10.1136/sextrans-2011-050187

Tsuchiya A, Brazier J, Roberts J. Comparison of valuation methods used to generate the EQ-5D and the SF-6D value sets. *J Health Econ*. 2006;25(2):334-346. doi:10.1016/j.jhealeco.2005.09.003

Van Den Hout WB, De Jong Z, Munneke M, et al. Cost-utility and cost-effectiveness analyses of a long-term, high-intensity exercise program compared with conventional physical therapy in patients with rheumatoid arthritis. *Arthritis Care Res*. 2005;53(1):39-47. doi:10.1002/art.20903

Van Der Pol M, Shiell A. Extrinsic goals and time tradeoff. *Med Decis Mak*. 2007;27(4):406-413. doi:10.1177/0272989X07302127

van Dongen JM, van denBerg B, Bekkering GE, van Tulder MW, Ostelo RWJG. Patient versus general population health state valuations: a case study of non-specific low back pain. *Qual Life Res*. 2017;26(6):1627-1633. doi:10.1007/s11136-017-1497-5

Van Dongen JM, Van Hooff ML, Finch AP, et al. Do socio-demographic characteristics and/or health status explain the magnitude of differences between patient and general public utility values? A chronic low back pain patients case study. *Health Qual Life Outcomes*. 2019;17(1):1-8. doi:10.1186/s12955-019-1240-8

Van Osch SMC, Stiggelbout AM. Understanding VAS valuations: Qualitative data on the cognitive process. *Qual Life Res*. 2005;14(10):2171-2175. doi:10.1007/s11136-005-6808-6

Vermeulen KM, Jansen DEMC, Buskens E, Knorth EJ, Reijneveld SA. Serious child and adolescent behaviour disorders; A valuation study by professionals, youth and parents. *BMC Psychiatry*. 2017;17(1):1-8. doi:10.1186/s12888-017-1363-6

Voormolen DC, Polinder S, von Steinbuechel N, et al. Health-related quality of life after traumatic brain injury: deriving value sets for the QOLIBRI-OS for Italy, The Netherlands and The United Kingdom. Qual Life Res. 2020;36:3095-3107. doi:10.1007/s11136-020-02583-6

Wang AMQ, Retrouvey H, Krahn M, McCabe SJ, Baltzer HL. Direct and indirect utilities of patients with mild to moderate versus severe carpal tunnel syndrome. J Hand Surg Eur Vol. 2020;45(8):832-837. doi:10.1177/1753193420922791

Wang SL, Hsieh E, Zhu LA, Wu B, Lu LJ. Comparative Assessment of Different Health Utility Measures in Systemic Lupus Erythematosus. *Sci Rep*. 2015;5:1-6. doi:10.1038/srep13297

Wang X, Zhuo L, Ma Y, et al. Similar responses to EQ-5D-3L by two elicitation methods: Visual analogue scale and time trade-off. *BMC Med Res Methodol*. 2020;20(1):1-10. doi:10.1186/s12874-020-01008-9

Wang Y, Xie F, Kong MC, et al. Patient-reported health preferences of anticoagulant-related outcomes. *J Thromb Thrombolysis*. 2015;40(3):268-273. doi:10.1007/s11239-015-1191-9

Webb EJD, O'Dwyer J, Meads D, Kind P, Wright P. Transforming discrete choice experiment latent scale values for EQ-5D-3L using the visual analogue scale. *Eur J Heal Econ*. 2020;21(5):787-800. doi:10.1007/s10198-020-01173-0

Webb EJD, Kind P, Meads D, Martin A. Does a health crisis change how we value health? Heal Econ (United Kingdom). 2021;30(10):2547-2560. doi:10.1002/hec.4399

Wee HL, Li SC, Xie F, et al. Are Asians comfortable with discussing death in health valuation studies? A study in multi-ethnic

Singapore. *Health Qual Life Outcomes*. 2006;4. doi:10.1186/1477-7525-4-93

Wildi SM, Cox MH, Clark LL, et al. Assessment of health state utilities and quality of life in patients with malignant esophageal dysphagia. *Am J Gastroenterol*. 2004;99(6):1044-1049. doi:10.1111/j.1572-0241.2004.30166.x

Williams K, Gibson A, McNamara L, Jones T, Lloyd AJ. Health state utilities associated with caring for an individual with cutaneous T-cell lymphoma (CTCL). J Med Econ. 2020;23(10):1142-1150. doi:10.1080/13696998.2020.1793764

Wong JB, Ramey DR, Singh G. Long-term morbidity, mortality, and economics of rheumatoid arthritis. *Arthritis Rheum*. 2001;44(12):2746-2749. doi:10.1002/1529-0131(200112)44:12<2746::AID-ART461>3.0.CO;2-Z

Worbes-Cerezo M, Nafees B, Lloyd A, Gallop K, Ladha I, Kerr C. Disutility Study for Adult Patients with Moderate to Severe Crohn's Disease. J Heal Econ Outcomes Res. 2019;6(2):47-60. doi:10.36469/9685

Wu XY, Ohinmaa A, Johnson JA, Veugelers PJ. Assessment of children's own health status using visual analogue scale and descriptive system of the EQ-5D-Y: Linkage between two systems. *Qual Life Res*. 2014;23(2):393-402. doi:10.1007/s11136-013-0479-5

Yang Z, Feng Z, Busschbach J, Stolk E, Luo N. How Prevalent Are Implausible EQ-5D-5L Health States and How Do They Affect Valuation? A Study Combining Quantitative and Qualitative Evidence. *Value Heal*. 2019;22(7):829-836. doi:10.1016/j.jval.2018.12.008

Yang Z, Luo N, Bonsel G, Busschbach J, Stolk E. Effect of Health State Sampling Methods on Model Predictions of EQ-5D-5L Values: Small Designs Can Suffice. *Value Heal*. 2019;22(1):38-44. doi:10.1016/j.jval.2018.06.015

Yi MS, Britto MT, Wilmott RW, et al. Health values of adolescents with cystic fibrosis. *J Pediatr*. 2003;142(2):133-140. doi:10.1067/mpd.2003.51

Yong YV, Shafie AA. Development, feasibility, and validity of a computer-based utility assessment tool for measuring asthma-specific health utilities in Malaysia using the standard gamble method. *J Asthma*. 2016;53(8):835-842. doi:10.3109/02770903.2016.1156694

Yu YR, Cunningham ME, DeMello AS, et al. Cost-Effectiveness Analysis of the Surgical Management of Infants Less than One Year of Age with Feeding Difficulties. *J Pediatr Surg*. 2020;55(1):187-193. doi:10.1016/j.jpedsurg.2019.09.076

Zhang XH, Xie F, Wee HL, Thumboo J, Li SC. Applying the expectancy-value model to understand health values. Value Heal. 2008;11(SUPPL. 1):S61-S68. doi:10.1111/j.1524-4733.2008.00368.x

Zijlstra TR, Braakman-Jansen LMA, Taal E, Rasker JJ, van de Laar MAFJ. Cost-effectiveness of Spa treatment for fibromyalgia: General health improvement is not for free. *Rheumatology*. 2007;46(9):1454-1459. doi:10.1093/rheumatology/kem157

Zrubka Z, Hermann Z, Gulácsi L, et al. Determinants of the acceptability of health problems in different ages: exploring a new application of the EQ VAS. *Eur J Heal Econ*. 2019;20(0123456789):31-41. doi:10.1007/s10198-019-01060-3

Table S3: Designs of the VAS used in the included studies (n = 308)

Designs of the VAS	n	%
EQ VAS		
EQ VAS	113	36.7
EQ VAS; vertical	14	4.5
EQ VAS; vertical, 10 cm	1	0.3
EQ VAS; vertical, 20 cm	14	4.5
EQ VAS; vertical, 50 cm	1	0.3
EQ VAS; horizontal	1	0.3
EQ VAS; horizontal, hashmarked, without numbers	1	0.3
EQ VAS; 10 cm	1	0.3
EQ VAS; 20 cm	3	1.0
EQ VAS; to give a number	2	0.6
EQ VAS and VAS PI	1	0.3
Unspecified VAS		
VAS	97	31.5
VAS; vertical	20	6.5
VAS; vertical, 20 cm	2	0.6
VAS; vertical, 25 cm	1	0.3
VAS; vertical, 50 cm	1	0.3
VAS; vertical, 55 cm	2	0.6
VAS; horizontal	4	1.3
VAS; horizontal, 10 cm	10	3.2
VAS; horizontal, 20 cm	1	0.3
VAS; 10 cm	14	4.5
VAS; 15 cm	1	0.3
VAS; 20 cm	1	0.3
VAS; 100 cm	1	0.3
EF-VAS	1	0.3

VAS Visual Analogue Scale; *EQ VAS* EQ Visual Analogue Scale; *VAS PI* Visual Analogue Pain Index; *EF-VAS* Erectile Function Visual Analogue Scale

Table S4: Types of anchoring for the VAS used in the included studies (n = 308)

Types of anchoring	n	%
Negative value or 0 to 100		
0 (worst imaginable health state) and 100 (best imaginable health state)	118	38.3
0 (worst imaginable health state) and 100 (perfect health)	2	0.6
0 (worst imaginable health state) and 100 (full health)	1	0.3
0 (dead/death) and 100 (perfect health)	49	15.9
0 (dead/death) and 100 (full health)	6	1.9
0 (death) and 100 (best imaginable health state)	1	0.3
0 (death) and 100 (best imaginable health state); 0 (worst imaginable health state) and 100 (best imaginable health state)	1	0.3
0 (death) and 100 (excellent or best health)	1	0.3
0 (death) and 100 (best health)	1	0.3
0 (death or worst possible health state) and 100 (best possible health state)	1	0.3
0 (very poor health state or death) and 100 (perfect health)	1	0.3
0 (immediate death) and 100 (normal, unburdened life)	1	0.3
0 (least desirable health state) and 100 (most desirable health state)	3	1.0
0 (least desirable health state) and 100 (most desirable health state; perfect health)	2	0.6
0 (least desirable health state) and 100 (perfect health)	1	0.3
0 (least preferred state) and 100 (most preferred state)	2	0.6
0 (worst possible health state) and 100 (best possible health state)	5	1.6
0 (worst possible health state) and 100 (full health)	2	0.6
0 (worst health state) and 100 (best health state)	4	1.3
0 (worst quality of life) and 100 (best quality of life)	1	0.3
0 (worst ranked state) and 100 (perfect health)	1	0.3
0 (no problems) and 100 (worst imaginable problems)	1	0.3
0 (really bad) and 100 (perfect)	1	0.3
0 and 100 (perfect health)	1	0.3
0 (death) and 100	1	0.3
0 and 100	3	1.0

0% (dead) and 100% (disease-free health)	1	0.3
0% (dead) and 100% (perfect health)	1	0.3
negative values (worse than death), 0 (death) and 100 (perfect health)	1	0.3

Negative value or 0 to 10		
0 (death) and 10 (perfect health)	1	0.3
0 (worst imaginable health state) and 10 (best imaginable health state)	1	0.3
0 (worst possible health or death) and 10 (perfect health)	1	0.3
0 (no quality of life at all) and 10 (optimal quality of life)	1	0.3
-10 (worst imaginable health), 0 (dead), 10 (full health)	1	0.3
0 to 1		
0 (death) and 1 (best imaginable health state)	2	0.6
0 (death) and 1 (best or normal health state)	1	0.3
0 (death) and 1 (perfect health)	5	1.6
0 (worst imaginable health state) and 1 (best imaginable health state)	4	1.3
0 (worst possible health state) and 1 (perfect health)	1	0.3
0 (worst possible health; as bad as or worse than death) and 1 (best possible health; perfect health)	1	0.3
0 (worst health state) and 1 (perfect health)	2	0.6
Anchors without numbers		
dead/death and perfect health	17	5.5
death and optimal health	1	0.3
worst imaginable health state and best imaginable health state	10	3.2
worst attainable health state and best attainable health state	1	0.3
extreme-level descriptors in each dimension	1	0.3
least important and extremely important	1	0.3
Not reported	42	13.6

VAS Visual Analogue Scale

Table S5: Reported advantages of using a VAS in the included studies

Quotations from the studies about advantages of using a VAS	References
Administration	
VAS is easy to understand.	Ock (2016)
VAS scoring task is simple and straightforward.	Shmueli (2008)
VAS is described as simple, easy to administer and reduce cost and time in data collection.	Wu (2014)
VAS is the simplest and most practical method of health state valuation that can be generally understood and answered by most individuals, including older children and adolescents.	Dillman (2016)
VAS is a simple measure to obtain and as a preference value, it is risk-free for the patient.	Moore (1999)
VAS method is quickly administered and is the most understandable and consistent way to elicit social values in the general Korean population.	Kim (2017)
VAS is practicable for Parkinson's disease (PD) patients as it avoids writing, which may be severely impaired in PD patients.	Demeulemeester (2015)
Scoring may be a more intuitive approach to valuation than drawing lines to the VAS.	Devlin (2005)
Comparison with other valuation methods	
VAS was an easier to understand method than the TTO or SG, which were both considered difficult.	Khabibullina (2019)
The rating scale is associated with less random measurement error as it is considerably easier to administer and understand than the TTO and the SG questions.	Lundberg (1999)

Respondents found TTO valuations to be more difficult than VAS valuations.	Yusof (2012)
Self-rated is easier (than TTO) for children to value.	Wu (2014)
Both the VAS and the TTO proved to be feasible and reliable for use in this sample of the Spanish general population, with the VAS implying lower respondent burden and administration costs and providing slightly higher reliability than the TTO.	Badia (1999)
TTO is a more reliable valuation technique, but VAS is more practical in use and is therefore an accepted technique for preference value measurement.	Cleemput (2010)
The trade of life years in TTO is culturally unacceptable compared to VAS.	Yusof (2012)
VAS values had better model fit compared to TTO values.	Yusof (2012)
A model using the VAS scores as dependent variables showed a good predictive ability and goodness of fit comparable to those of the TTO model.	Cho (2015)
VAS appears to be more variable and sensitive to symptoms than the SG. VAS appears to be more suitable for studies that examine the impact of an intervention on the population with cirrhosis.	Foster (2020)
Applications	
VAS is feasible and acceptable.	Wu (2014)
VAS can serve as a feasible and reliable method to elicit patient values and is the predominant basis for generating an experienced health state-based value set.	Pickard (2017)
VAS method appears to be a more valid approach for valuation in the general population due to its greater simplicity and feasibility.	Wang (2020)

That the rating scale method is most strongly related to the dimensions of quality of life could be used as an argument for using the rating scale method to measure health-state utilities.	Lundberg (1999)
VAS is not a multi-attribute, choice-based preference instrument, but it was included because it is sometimes seen as a more practical and equally valid alternative.	Langfitt (2006)
This study has demonstrated a high degree of acceptance by respondents and suggests that the VAS method can perform very successfully in generating health state valuations from the general public. These are powerful arguments for the serious consideration of VAS in a survey context, possibly alongside a choice-based method. Given its ease of use and low resource cost, it is time to reconsider the place of VAS (and other category rating methods) in the study of health.	Gudex (1996)
VAS gives a single score where health states lie compared to the best and worst imaginable overall health states.	Zrubka (2019)
VAS reflects the general health status of a patient with lupus.	Moore (1999)
VAS can be used as a ‘‘stand-alone’’ question, without any need for recall health data to be invoked.	Shmueli (2008)
Based on our findings, VAS captures the variability of utility across various pregnancy contexts and may be the most appropriate metric for use in assessing health utility among pregnant women.	Lundsberg (2017)
The stability found indicates that the VAS is quite robust with respect to growing exposure to health details.	Shmueli (2008)
The results of our study show that subject-reported VAS is sensitive to changes in HRQOL– health state.	Dillman (2016)

EQ VAS may incorporate health information not reflected in the EQ-5D.	Chapman (2009)
In the EQ VAS assessment, participants included all important health aspects that they perceived were not covered by the EQ-5D-5L descriptive system, such as stress, well-being, relationships, job satisfaction, and fitness.	Ernstsson (2020)
VAS terms are also likely to be proxying for effects not otherwise represented in the model.	Dolan (2002)
VAS performs better in a predictive capacity in that those patients who require fewer visits have, on average, higher initial VAS scores.	Dolan (1999)
VAS is a simple and effective tool for economic evaluations.	Yu (2020)
VAS is easy to administer, quick and cheap, and can be easily used in decision-making models in everyday practice.	Loon (2017)
VAS might have an important role in familiarizing respondents with health states and with the idea that they can be expressed using a scale.	Dolan (2002)

VAS Visual Analogue Scale

Table S6: Reported disadvantages of using a VAS in the included studies

Quotations from the studies about disadvantages of using a VAS	References
Comparison with other valuation methods	
VAS measures attributes similarly to other generic health instruments (nonutility based), but TTO and SG incorporate preferences in a way that may better reflect decision-making processes.	Suarez-Almazor (2001)
Similar to the direct utility instruments (standard gamble and time trade-off) the Euro-thermometer VAS does not evaluate specific quality of dimensions but is not preference-based.	Huber (2021)
VAS is different from the SG, TTO, and WTP, in that it does not involve any tradeoffs or personal attitudes toward risk, time, or money.	King (2004)
An important consequence of the compression of VAS values is that the TTO is likely to discriminate better between health states at many points on the health continuum.	Badia (1999)
Only 3 of the instruments (the SF-6D, the Euro-thermometer VAS, and the time trade-off test) were responsive to CPAP treatment, while 2 (including the widely used EQ-5D and the standard gamble test) remained unchanged despite major improvements in breathing disturbances and symptoms.	Huber (2021)
The selection of VAS value set goes against the current preference of TTO based value sets in Asia.	Yusof (2012)
The VAS and SG-tool received similar difficulty ratings.	Yong and Shafie (2016)
Applications	
VAS rating scale represents a preference-based method but lacks the equivalent conceptual richness and does not reflect how people actually form their preferences.	Khabibullina (2019)

The limitation of employing VAS-rated health states is that VAS methods are not regarded to measure preference on a cardinal scale.	Seidl (2015)
Responses to rating scales, including the VAS, tend not to have interval scale properties because no trade-offs are expressed.	Krabbe (2017)
VAS does not measure utilities, as only choice-based measurement techniques allow for the measurement of utilities.	Cleemput (2010)
VAS is not a choice-based method and there is a lack of choice under uncertainty.	Wu (2014)
VAS does not involve any sacrifice, and the severity of other health states being valued has been shown to influence the values produced.	Shafie and Thakumar (2020)
As the VAS does not incorporate a risk or a trade-off, it may lead to systematically lower scores.	Kiebert (2001)
Although the single-item VAS is convenient, utility preference scores from the VAS do not appear to be valid assessments of HRQOL in women with pelvic floor disorders.	Harvie (2018)
Although the single item VAS is convenient, utility preference scores from the VAS do not appear to be valid assessments of HRQOL in women with urinary incontinence.	Harvie (2014)
Although the single-item VAS appears to be convenient, it does not appear to produce scores that are valid assessments of utilities in women with fecal incontinence.	Harvie (2011)
Although median utilities for the CSG and VAS were higher, rather than lower, in the severe group, this was not significant, suggesting that the CSG and VAS did not have	Wang (2020)

enough sensitivity to distinguish between these two levels of severity.	
The VAS method is not considered a real preference-measurement instrument because it does not require participants to consider the potential downsides of therapy.	Faris (2018)
VAS is prone not only to end-aversion but also to context or reference bias.	Krabbe (2017)
VAS does not appear to add very much to the explanatory power of the model.	Dolan (2002)
VAS showed lower correlation with other generic HRQOL instruments (HUI-3, EQ-5D, and SF-6D) as well as condition-specific symptom severity and quality-of-life instruments (the PFDI and PFIQ).	Harvie (2011)

VAS Visual Analogue Scale

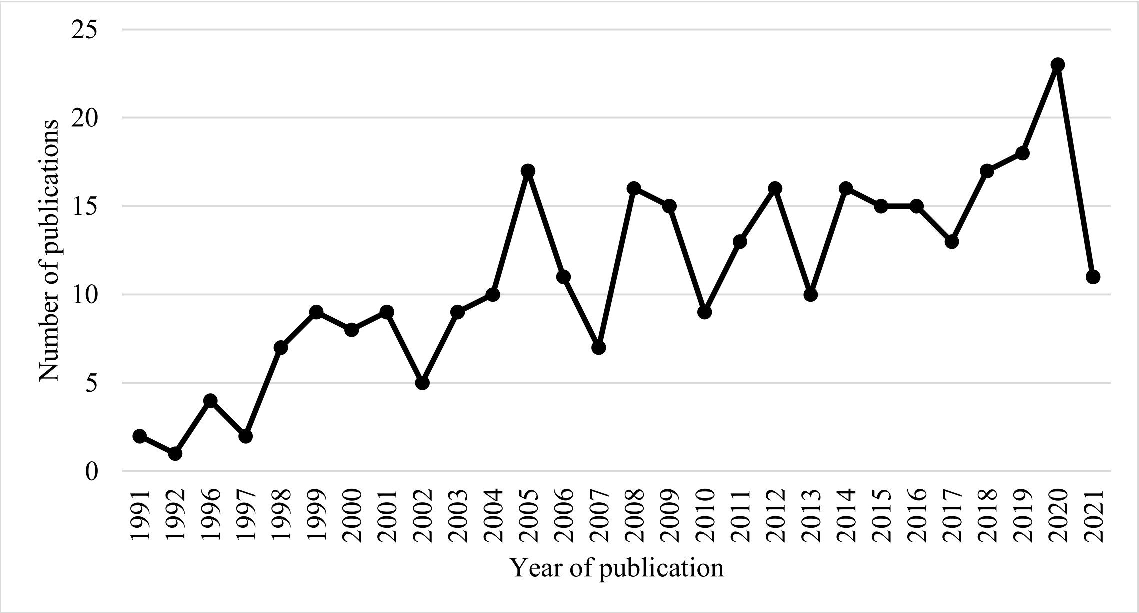

Figure S1: Number of Included Studies by Publication Year (n = 308)

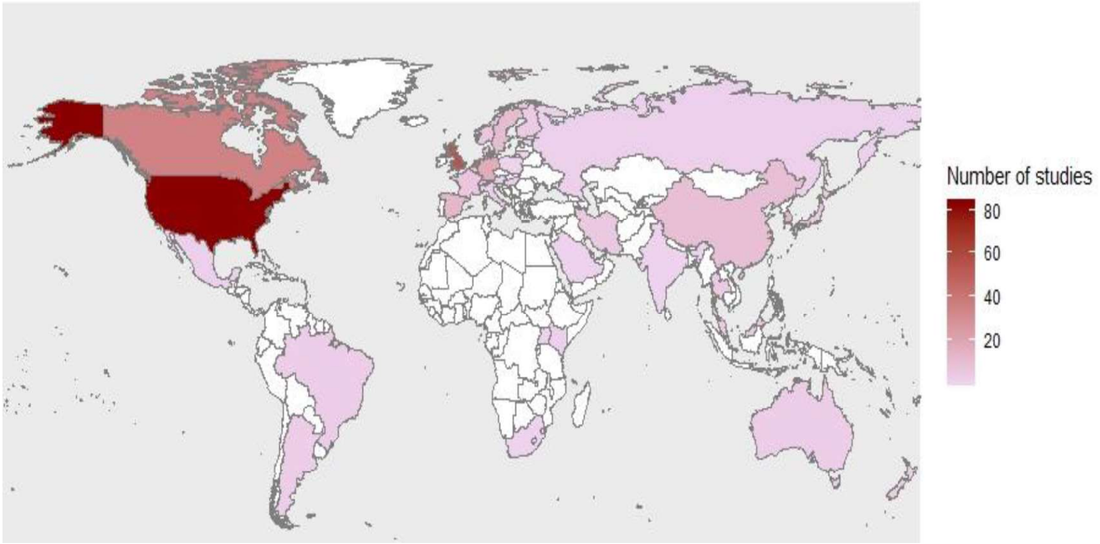

Figure S2: Countries Where the Included Studies Were Conducted (n = 308)
